# Living with breathing pattern disorder: a scoping review

**DOI:** 10.1038/s41533-026-00495-5

**Published:** 2026-03-25

**Authors:** Catherine Moffat, Susan Walker, Jonathan Fuld, Shanlee Higgins

**Affiliations:** 1 Anglia Ruskin University, Faculty of Health, Medicine and Social Care, Cambridge, UK; 2 Breathlessness Intervention Service, Department of Palliative Care, Cambridge University Hospitals NHS Foundation Trust, Cambridge, UK; 3 Department of Respiratory Medicine, Cambridge University Hospitals NHS Foundation Trust, Cambridge, UK; 4 Victor Phillip Dahdaleh Heart and Lung Research Institute, University of Cambridge, Cambridge, UK

**Keywords:** Diseases, Health care, Medical research, Psychology, Psychology, Signs and symptoms

## Abstract

A scoping review was conducted using the Arksey and O’Malley framework to develop a comprehensive overview of how the experience of breathing pattern disorder (BPD) has been conceptualized and reported in the literature. The Applied Social Sciences Index and Abstracts (ASSIA), Cumulative Index to Nursing and Allied Health Literature (CINAHL), Embase, Medline and PsycINFO electronic databases were searched from the earliest available reports until 13^th^ May 2025. The reference lists of included reports were also screened for appropriate literature. Only reports relating to adults and published in English were included. Sixty-two reports were included in the final review. The following eight themes emerged; 1: Symptoms were diverse and frequently mimicked serious pathology. 2: Symptom triggers were often stress related. 3: Precipitating life experiences for BPD included physical or psychological trauma, chronic anxiety or stress. 4: The diagnostic pathway was often prolonged and complex. 5: Patient reaction to diagnosis varied from welcoming, sceptical or rejection. 6: Healthcare use was frequent due to symptoms mimicking serious pathology and a prolonged diagnostic pathway. 7: BPD was associated with panic, anxiety, fear, phobias and depression. 8: BPD was also associated with reduced quality of life and poorer asthma control. Evidence for the experience of BPD is poorly represented in the literature, mainly viewed from a Western perspective and found in simple case reports over 25 years old and snippets within empirical studies. Literature searching was confounded by the change in terminology over time, and the lack of agreed definition and diagnostic methods for BPD. Further research is required employing more diverse and rigorous study designs to clarify the definition and diagnostic methods for the condition, as well as explore experience of BPD, including experience from outside Europe and North America.

## Introduction

Breathing pattern disorder (BPD) is a contemporary healthcare issue, with an incidence of around 9.5% in the adult population, occurring in 14% of females and 2% of males^1^. It is less prevent in adolescents occurring at a rate of 2.5%^2^, and in children with asthma at a rate of 5.3%^3^. BPD is often a co-morbidity, rather than a standalone pathology, with a higher incidence in chronic obstructive pulmonary disease (COPD) of 47%^4^, 17.6–64% in asthma^5^ and 29.4–63.4% in post-coronavirus disease (COVID) syndrome^6,7^. BPD is also prevalent in elite athletes with an incidence of 22%^8^ and has been found in postural orthostatic tachycardia syndrome (POTS)^9^ and implicated in musculoskeletal pain and movement disorders^10,11^.

BPD is a condition in which the breathing pattern is no longer efficient, appropriate, adaptive or responsive to activity, leading to symptoms such as breathlessness, unable to take a satisfying breath in, frequent sighing and chest pain^12^. The pathogenesis is unclear, however a combination of psychophysiological, biomechanical and biochemical factors have been suggested^13^. The condition remains poorly understood with no accepted terminology, definition or diagnostic method^13–17^ although Barker and Everard (2015)^12^^, p.53^ propose ‘an alteration in the normal biomechanical patterns of breathing that result in intermittent or chronic symptoms which may be respiratory and/or non-respiratory.’ Common assessment tools currently used in clinical practice to aid diagnosis or act as outcome measures include the Nijmegen Questionnaire (NQ), Breathing Pattern Assessment Tool (BPAT), Short Evaluation of Breathing Questionnaire (SEBQ), Manual Assessment of Respiratory Motion (MARM), Cardiopulmonary Exercise Testing (CPET) and breath hold^15^^,^^17–24^. Although most of these lack sufficient evidence or are perceived as complex to use^17^, with the NQ based only on the hyper-ventilatory form of BPD and not designed as a standalone diagnostic tool^25^. Subjective and objective assessment tools are often used in combination, as recommended by van Dixhoorn and Folgering (2015)^25^. Breathing retraining is the mainstay of treatment which may also involve relaxation and musculoskeletal techniques^13,14,26^.

Hyperventilation; ventilation in excess of metabolic requirements^27^, was the first type of BPD to be recognised, with Kerr, Gliebe and Dalton (1938)^28^ first using the term ‘hyperventilation syndrome’ (HVS). Later, Thomas et al. (2001)^29^, introduced the term ‘dysfunctional breathing’ implying other breathing patterns may be present which may not have hyperventilation as a feature. Following on from this Boulding et al. (2016)^30^, suggested that HVS was a subtype of BPD and proposed additional subtypes including thoracic dominant, forced expiratory, thoraco-abdominal asynchrony and periodic deep sighing. While Baker and Everard (2015)^12^ suggest classifying BPD in terms of cause, rather than presentation, proposing thoracic and extra thoracic, with extra thoracic including upper airway involvement, with each of these classifications further split into functional or structural in nature. In this scoping review the term BPD is used throughout to represent all subtypes of the condition.

BPD is complex in presentation, with varied symptoms across many body systems, including breathlessness and chest pain, which may mimic severe pathology. Patients with BPD are therefore frequently referred for a battery of cardiorespiratory investigations, before being told that they have no serious illness. Patient experience of this poorly understood condition has been over-looked, with a preliminary exploratory search of the literature revealing no previous reviews addressing this topic. A scoping review is therefore an appropriate starting point to create a comprehensive overview, mapping and synthesizing the breadth of available evidence to gain insight into this previously unexplored area. This exploratory work aims to identify the key characteristics of BPD experience, as well as types of evidence and knowledge gaps, aligning with scoping review purpose as defined by Munn et al.^31^.

## Methods

### Protocol and registration

The scoping review was conducted using the Arksey and O’Malley (2005)^32^ framework to explore the question ‘How has the experience of breathing pattern disorder been conceptualized and reported in the literature?’ The preferred reporting items for systematic reviews and meta-analyses extension for scoping reviews (PRISMA-ScR) methodology framework^33^ was adopted for presenting the results, however a registered protocol was not created. No funding was received for this review.

### Eligibility criteria

Only literature on adults was selected as BPD is more common in adults so the findings would be of interest to a larger audience clinically. The publication date was not restricted to ensure all relevant reports were included. Only literature published in English was included, due to limited time and resources, as this was the language spoken by the authors. Only sources published in full in scientific journals or as a conference abstract were included to promote confidence in the findings. The eligibility criteria can be found in Table 1.

**Table 1:** Eligibility criteria.

Inclusion criteria	Exclusion criteria
BPD experience as a stand-alone syndrome or as a comorbidity. Precipitating experience. Experience of the diagnostic pathway. Psychological factors linked to BPD. Effect of BPD on quality of life.	Paediatrics* Not published in English language. BPD not formally diagnosed. Organic cause of BPD. Voluntary hyperventilation. Sleep disordered breathing. No data on BPD experience.

*Paediatric is defined in Medline, CINAHL and PsychInfo as 18 years or younger. In ASSIA and Embase it is defined as 17 years or younger.

### Information sources

A database search was carried out in March 2022 and updated on the 13th May 2025. Databases Applied Social Sciences Index and Abstracts (ASSIA), Cumulative Index to Nursing and Allied Health Literature (CINAHL), Embase, Medline and PsycINFO were searched for the appearance of the search terms within the title or abstract of reports. The reference lists of included reports were also screened for relevant literature.

### Search

In preliminary searches ‘experience’ was included as a search term, however no articles were found. This term also introduced a possible bias towards qualitative literature. The search term ‘experience’ was therefore removed to capture a broader range of literature. Index terms were considered but no appropriate ones found. The search focused on finding studies relating to adults (population), diagnosed with BPD (concept), in any context anywhere in the world (context). ‘Adult’ was determined by the exclusion of paediatrics as defined in the eligibility criteria given in Table 1. As there is no agreed ‘gold standard’ BPD diagnostic method, diagnosis was via the method determined by the authors of each report.

The definition and terminology of BPD has changed over the years, making literature searching complex, therefore synonyms for BPD were included as search terms, with liberal use of the ‘wildcard’ function to allow for various endings. The Boolean operator ‘NOT’ was used to exclude reports containing the words ‘sleep’ or ‘nocturnal’ to remove reports regarding sleep disordered breathing, as this is a different condition from BPD. The final search terms are given in Table 2 and a full electronic search strategy for each database is provided in the supplementary material.

**Table 2:** Search terms.

Database	Search Terms
ASSIA, CINAHL, Medline, PsycINFO	“chronic hyperventilation” OR “hyperventilation syndrome*” OR “dysfunctional breathing” OR “breathing pattern dysfunction*” OR “disordered breathing” OR “breathing pattern disorder*” OR “idiopathic hyperventilation”
Embase	(chronic ADJ hyperventilation) OR (hyperventilation ADJ syndrome$) OR (dysfunctional ADJ breathing) OR (breathing ADJ pattern ADJ dysfunction$) OR (disordered ADJ breathing) OR (breathing ADJ pattern ADJ disorder$) OR (idiopathic ADJ hyperventilation)

*ASSIA* Applied Social Sciences Index and Abstracts, *CINAHL* Cumulative Index to Nursing and Applied Health Literature.

### Selection of sources of evidence

BPD had to be formally diagnosed. Reports that focused on organic causes of BPD, including sleep disordered breathing were excluded, as was voluntary hyperventilation. Reports that did not include any information on experience were also excluded, as were reports that focused on paediatrics. A preferred reporting items for systematic reviews and meta-analyses (PRISMA) diagram^34^ was used to document the selection process.

### Data charting process

The title and abstracts of retrieved reports were screened using the eligibility criteria in Table 1. Database searching, report screening and data extraction were carried out by the lead author alone as this scoping review originated as part of a doctorial study, and as such was peer reviewed by members of the doctorial school faculty. Data extraction was crossed checked by the lead author and a selection of papers were also checked by each of the other authors for accuracy. A data extraction table was used to capture the themes and is provided in the supplementary material.

### Data items and synthesis of results

Retrieved reports were screened for data on experience of BPD. Initial data extraction focused on the themes of symptoms, episodic triggers, precipitating experience and diagnostic pathway based on preliminary searching, reading and clinical experience. Additional themes of healthcare use, reaction to diagnosis, psychological factors and quality of life were added as these became apparent. All themes were created and viewed through the lens of the person with BPD, with data extracted to capture the experience of living with the condition. Precipitating experience included significant life events prior to BPD onset, as well as significant ongoing life experience noted in the literature. Quality of life was captured broadly, including formal quality of life measures, as well as other factors which may impact the quality of a person’s life experience. Critical appraisal of individual sources of evidence was not carried out. A table was created to summarise the key features of the included reports, as well as the data found within the themes.

## Results

**Fig. 1:**
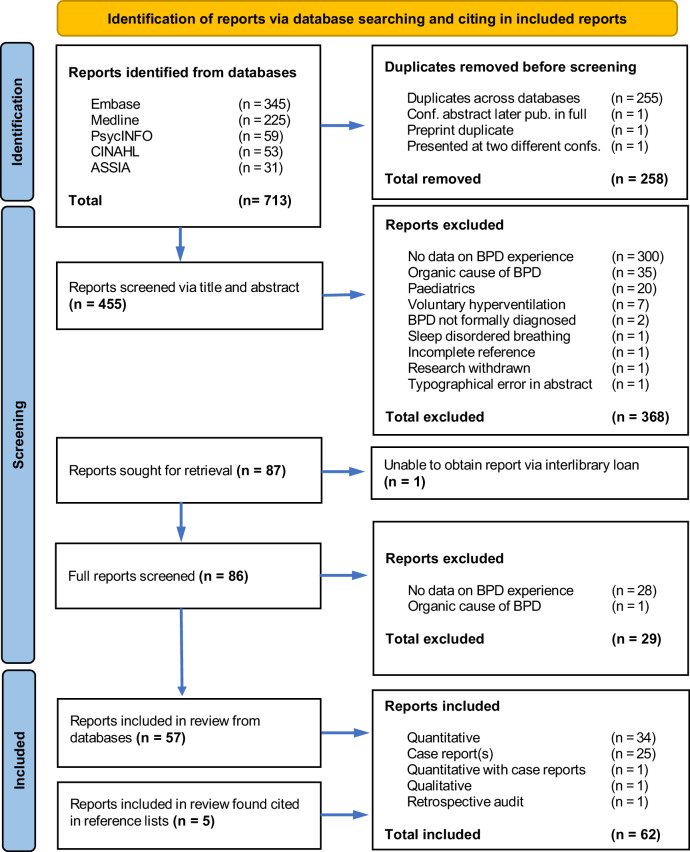
PRISMA Diagram. ASSIA Applied Social Sciences Index and Abstracts, CINAHL Cumulative Index to Nursing and Allied Health Literature, BPD Breathing Pattern Disorder. Adapted from (Page, M. J. et al.)^34^.

Seven-hundred and thirteen reports were identified, of which 258 were duplicates, leaving 455 for screening. After screening for the eligibility criteria in Table 1, 368 reports were excluded, leaving 87 for retrieval. The full text of one report was unobtainable. The full text of 86 reports were screened, with 29 excluded on reviewing the full report, leaving 57 reports to be included in the final review. An additional five reports were included which were found cited in reference lists during the screening process, bringing the total to 62 reports included in the final review. The report selection process is illustrated in PRISMA diagram (Fig. 1). Key characteristics of the included reports and data extracted is presented in Table 3.

**Table 3:** Characteristics of included reports and themes relating to BPD experience.

Reference	Report type	BPD diagnostic method(s)	Population from which participants/patients were recruited and inclusion diagnosis (if applicable)	Location of report	Funding	Themes relating to BPD experience
Agache et al. (2012)^81^	Quantitative	NQ, CPET	Referred to allergy and clinical immunology clinic. Asthma diagnosis.	Romania	Theramed Medical Centre.	Anxiety (PS) BPD related to severe, brittle asthma and frequent exacerbations, GERD, rhinitis (QOL)
Bass and Gardner (1985)^45^	Quantitative	Capnography at rest, during exercise and during voluntary hyperventilation. ABG for some participants.	Patients referred to ‘specialist physicians’ (clinical speciality not specified).	UK	Not disclosed.	Cardiorespiratory, neurological (S) Surgery, possible pulmonary embolism (PE) Exhaustive investigations NAD (DP) Anxiety, panic, phobias, agoraphobia (PS)
Blau, Wiles and Solomon (1983)^36^	Case report(s), letter to editor	HVPT	Referred to neurology clinic.	UK	Not disclosed.	Cardiorespiratory, neurological, visual, gastrointestinal, other (S) Unable to find employment, employment stress (PE) Neurological investigations, accused of malingering by HCP (DP) Anxiety (PS)
Bonde et al. (2013)^48^	Quantitative	Not specified	General population.	Sweden	Not disclosed.	Cardiorespiratory, MSK (S) Mental stress or conflict (ET)
Brashear (1984)^49^	Case report(s), discussion paper	Not specified	Referred to respiratory clinic.	USA	Not disclosed.	Cardiorespiratory, visual, neurological (S) Bereavement (PE) Hospitalisation (HU) Unable to leave apartment (QOL)
Brodtkorb et al. (1990)^37^	Quantitative	HVPT with capnography	Referred to neurology clinic.	Norway	Not disclosed.	Neurological, MSK, ENT, visual, psychological (S) Mental stress (ET) Conflict, stress, financial concerns, relationship issues (PE) Neurological investigations, suspected various neurological diagnoses (DP) Participants lacked insight into psychological basis of symptoms (DR) Hospital admissions for pseudoseizures (HU) Depression (PS)
Byrne, Pfeffer and De Simoni (2023)^78^	Qualitative	Self-report of asthma with ILO or BPD.	Review of social media posts (Asthma and Lung UK community platform on Health Unlocked) Self-report of asthma with ILO or BPD.	UK based online platform with worldwide posts.	National Institute for Health and Care Research Program Grant for Applied Research.	Respiratory (S) Misdiagnosed as asthma, accused of hypochondria, delayed diagnosis (DP) Positive participant response to diagnosis. Participants sceptical at first to diagnosis. Participant felt blamed or criticised by HCPs. Negative response by HCPs. HCPs lacked awareness of BPD (DR) A & E visits, hospital admissions (HU)
Castro et al. (2000)^75^	Case report(s), letter to editor	ABG	Referred to cardiology clinic.	Chile	Not disclosed.	Cardiorespiratory, neurological, psychological (S) Cardiorespiratory, neurological investigations (DP) Hospital admission (HU)
Chenivesse et al. (2014)^87^	Quantitative	NQ, CPET, HVPT with capnography	Referred to tertiary breathlessness clinic.	France	No funding received.	Impaired health related QOL all domains, particularly physical functioning and role limitation as a result of physical problems. Health related QOL much lower when compared with COPD, severe asthma, cystic fibrosis, social anxiety and panic disorder (QOL)
Cherif et al. (2024)^73^	Quantitative	NQ	Previous COVID-19 pneumonia.	Tunisia	No funding received.	Severe or ‘critical’ COVID-19 infection (PE) Post traumatic stress disorder (PS)
Chevalier and Schwartzstien (2000)^50^	Case report(s), discussion paper	RR, V_E_, ABG, CPET	Not specified.	USA	Not disclosed.	Cardiorespiratory, neurological, ENT (S) Pneumonia, childhood abuse (PE) Multiple cardiorespiratory investigations NAD (DP) Depression (PS)
Conway, Freeman and Nixon (1988)^68^	Quantitative	HVPT with capnography	Referred to service (clinical speciality not specified).	UK	Research Council for Complementary Medicine. Charing Cross Hospital Governor’s Fellowship.	Bereavement following the death of a close family member, rejection, loss of physical control, anger (PE) Fear of snakes, anxiety about health (PS)
Decuyper et al. (2012)^74^	Quantitative	NQ, HVPT with TcPCO_2_ monitoring	Referred to psychological department.	Belgium	Not disclosed.	Cardiorespiratory, neurological, ENT, MSK, gastrointestinal, visual, other (S) No organic disease found, BPD diagnosed by exclusion (DP)
Denton, Bondarenko and Hew (2018)^90^	Quantitative, conference abstract	NQ	Undergoing physiotherapy breathing retraining in a difficult asthma clinic. Asthma diagnosis.	Australia	National Health and Medical Research Council, Australia.	BPD associated with poor asthma control and poor asthma related QOL (QOL)
Evans (1995)^38^	Case report(s), discussion paper	HVPT	Referred to neurology clinic.	USA	Not disclosed.	Cardiorespiratory, neurological, other (S) Symptoms during work meetings (ET) Cardiorespiratory, neurological investigations. BPD misdiagnosed as partial seizures (DP) Patient unconvinced by diagnosis, wanted further imaging to rule out other causes (DR) Depression, fear of redundancy (PS)
Freeman, Conway and Nixon (1986)^62^ *(Focus on EtCO_*2*_*)*	Quantitative	Psychological challenge under hypnosis and HVPT both with capnography	Referred to cardiology clinic.	UK	Research Council for Complementary Medicine. Charing Cross Hospital Governor’s Fellowship.	Neurological, MSK (S) Strong emotions, exercise, anger at boss (ET) Bereavement, physical trauma, psychological trauma (PE) Cardiorespiratory investigations (DP) Fear of snakes, claustrophobia, fear of rejection (PS)
Freeman, Conway and Nixon (1986)^70^ *(Focus on HR)	Quantitative	Psychological challenge under hypnosis and HVPT both with capnography	Referred to cardiology clinic.	UK	Research Council for Complementary Medicine. Charing Cross Hospital Governor’s Fellowship.	Cardiorespiratory, MSK (S) Repeated resuscitation attempts, discovery of a dead body, anger (PE)
Gardner and Bass (1989)^35^	Case report(s), discussion paper	HVPT with capnography	Two populations: Referred to respiratory clinic. Admission to psychiatric hospital.	UK	Not disclosed.	Cardiorespiratory, neurological (S) Heart murmur as a child (PE) Psychiatric hospital admission (HU) Panic attacks, anxiety about health- serious heart condition (PS) Incapacitated for 2-3 days (QOL)
Gilbert (1998)^63^	Case report(s), discussion paper	Not specified	Not specified.	USA	Not disclosed.	Cardiorespiratory, neurological, MSK (S) Trapped in a lift, bereavement following the death of a family member (PE)
Goff and Gaensler (1969)^40^	Case report(s), discussion paper	Exercise test with ABG	Admitted to hospital.	USA	National Heart Institutes, United Staes Public Health Service. Massachusetts Tuberculosis and Health League Inc.	Cardiorespiratory, neurological, MSK (S) Intrafamily conflict (PE) Cardiorespiratory, and neurological investigations NAD (DP) Two hospital admissions (HU) Anxiety (PS)
Greene (1984)^44^	Case report(s), discussion paper	HVPT with capnography	Referred to speech and language therapy.	UK	The Smith’s Charity.	Cardiorespiratory, neurological, ENT, MSK, visual, gastrointestinal (S) Presenting at business meetings, symptoms context specific (ET) Martial issues, stress of looking after other family members, domestic abuse, sense of failure or inadequacy, suppression of emotions (PE) A & E attendance (HU) Anxiety, panic attacks, fear of flying, agoraphobia, fear of redundancy (PS) Fainting and falling at work (QOL)
Grossman and De Swart (1984)^93^	Quantitative	HVPT with capnography	Referred for pulmonary function testing.	Netherlands	Not disclosed.	Cardiorespiratory, neurological, psychological, other (S)
Hagman, Janson and Emtner (2008)^57^	Quantitative	Ten point subjective and objective criteria developed by the authors.	Referred to lung and allergy clinic. Asthma diagnosis.	Sweden	Centre for Clinical Research Dalarna, Sweden.	Misdiagnosed as asthma (DP) More frequent A & E visits with breathing problems than those with asthma (HU) More anxious, depressed and impacted more by stress than those with asthma alone (PS) Lower health related QOL, especially social function and role emotion. More negatively affected by stress and breathing problems had greater impact on daily lives than asthma alone (QOL)
Han et al. (1998)^51^	Quantitative	NQ, HVPT with capnography	Referred for pulmonary function testing.	Belgium	Not disclosed.	Cardiorespiratory, neurological, gastrointestinal, psychological, MSK, visual, other (S)
Hegel et al. (1989)^41^	Case report(s) discussion paper	HVPT	Referred to behavioural medicine service.	USA	Not disclosed.	Cardiorespiratory, neurological (S) Cardiorespiratory, neurological, gastrointestinal investigations. Misdiagnosed as angina, possible heart failure (DP) Multiple A & E attendances (HU) Panic attacks (PS) Activity limited by chest pain (QOL)
Hoes et al. (1987)^65^	Quantitative	NQ, HVPT capnography, hypercapnic challenge, ABG, respiratory rate >10, irregular breathing pattern	Referred to psychiatry clinic. BPD with and without panic disorder.	Netherlands	Not disclosed.	Panic disorder (PS)
Howell (1990)^39^	Case report(s) series, discussion paper	HVPT with capnography	Referred to outpatient clinics with disproportionate breathlessness.	UK	Not disclosed.	Cardiorespiratory, neurological (S) Symptoms at rest when relaxed (ET) Bereavement, separation, resentment, marital disharmony, living alone, previous surgery (PE) Patients more readily accepted diagnosis if present as physical condition rather than linked to anxiety (DR) Anxiety, depression, anxiety about health (PS)
Huey and West (1983)^52^	Quantitative	Authors created a hyperventilation screening questionnaire based on common symptoms related to BPD given in the literature. Minute ventilation, RR, HVPT	Students enrolled on a psychology course.	USA	Arizona State University.	Cardiorespiratory, neurological, MSK (S)
Kang (2015)^66^	Case report(s), discussion paper	ABG	Presented to accident and emergency.	Korea	No funding received.	Neurological (S) Physical assault (PE) Cardiorespiratory, neurological investigations (DP) A & E attendance, hospital admission (HU)
Kerr et al. (1937)^58^	Case report(s) discussion paper	HVPT	Not specified.	USA	Not disclosed.	Cardiorespiratory, neurological, MSK, gastrointestinal, ENT, psychological, other (S) Family disharmony, excitement, emotional distress, conflict, alcohol, being in public, prolonged laughing or talking (ET) Bereavement, surgery, marital or family disharmony, health or financial worries, divorce, witnessing an RTA, murder of family members, surgery, caring for a loved one at end of life (PE) Cardiorespiratory, neurological, gastrointestinal investigations NAD (DP) Depression, fear of being murdered, fear of husband dying, fear an illicit relationship will be discovered, anxiety about health (PS)
Kerr, Gliebe and Dalton (1938)^28^	Case report(s), discussion paper	HVPT	Not specified.	USA	Not disclosed.	Cardiorespiratory, neurological, ENT, gastrointestinal, other (S) Misdiagnosis (DP) Anxiety about health (PS)
King (1984)^64^	Case report(s), discussion paper	Not specified	Referred to cardiology clinic.	USA	Not disclosed.	Cardiorespiratory, neurological, ENT (S) Noise and bustle (ET) Work and marital problems (PE) Cardiorespiratory investigations, coronary artery disease suspected (DP) Anxiety, depression (PS) Unable do housework or most of cooking (QOL)
Koniukhovskaia (2022)^82^	Quantitative, Conference Abstract	NQ	General ‘healthy’ population.	Russia	Russian Scientific Foundation.	Association between BPD and high and borderline levels of situational and personal anxiety during the COVID-19 pandemic (PS)
Lapperre et al. (2020)^84^	Quantitative, conference abstract	NQ, body plethysmography	From the BREATHE study. COPD diagnosis.	Not disclosed	Not disclosed.	Higher anxiety and depression than COPD alone (PS) Poorer COPD related QOL than COPD alone (QOL)
Lazarus and Kostan (1969)^61^	Case report(s), discussion paper	HVPT	Psychiatric clinic.	USA	Not disclosed.	Cardiorespiratory, neurological, gastrointestinal (S) Argument with husband (ET) RTA, murder of close friend, marital disharmony, bereavement, traumatic anaesthetic experience (PE) A & E attendance, psychiatric hospital admission (HU) Anxiety, fear of the dark or being alone, fear of breakdown in relationship with husband, anxiety about health (PS)
Loew et al. (2022)^53^	Quantitative, conference abstract	Symptoms, CPET	Referred to a ‘Long COVID’ clinic. Post- COVID syndrome diagnosis.	Not disclosed	Not disclosed.	Cardiorespiratory (S) High burden of symptoms, functional impact and low QOL (QOL)
Magarian (1982)^54^	Case report(s), discussion paper	HVPT	Referred to general medicine clinic.	USA	HEW (acronym not explained).	Cardiorespiratory, neurological, ENT, gastrointestinal, psychological, MSK, other (S) Isolation, physical trauma, unemployment, wife requiring psychiatric care (PE) Cardiorespiratory investigations, heart failure suspected (DP) Hospital admissions (HU) Depression, nervousness, anxiety about health (PS)
Magarian and Olney (1984)^46^	Case report(s), discussion paper	HVPT	Referred to general medicine clinic.	USA	HEW (acronym not explained).	Cardiorespiratory, neurological, visual, other. Symptoms not in keeping with activity (S) Symptoms could occur at rest but could dance for hours (ET) Cardiorespiratory, neurological investigations NAD. Multiple neurological and cardiac misdiagnoses. Accused of malingering (DP) Multiple hospital admissions, clinic appointments, A & E attendances (HU) Work and home life affected (QOL)
Maltinsky et al. (2025)^86^	Quantitative	‘A clinical diagnosis of BPD’	Referred to two respiratory physiotherapy clinics. BPD diagnosis.	UK	Engineering and Physical Sciences Research Council.	Illness perception of people with BPD were negative, with the stronger belief that BPD was a serious condition, the more negative their mood and the greater the severity of anxiety and depression (PS) Participants believed that BPD had a substantial life impact (QOL)
Mooney and Candy (2008)^79^	Case report(s), conference abstract	NQ	Not specified.	Australia	Not disclosed.	Three A & E attendances in 10 days. (HU) 12 days sick leave (QOL)
Nguyen et al. (1992)^69^	Case report(s), discussion paper	HVPT, ABG	Presented to local hospital.	USA	Not disclosed.	Cardiorespiratory (S) Financial and martial difficulties (PE) Cardiorespiratory investigations. BPD misdiagnosed as asthma (DP) Relieved to be diagnosed with BPD and not chronic life threatening asthma (DR) ICU admission for suspected asthma (HU)
Noehren (1966)^47^	Case report(s), discussion paper	ABG	Admitted to hospital. COPD diagnosis.	USA	Not disclosed.	Cardiorespiratory, other. Inconsistent symptoms (S) Emotional stress (ET) Frequent pneumonia, favourite uncle died of pneumonia, mother died of pneumonia, father died heart disease. Several surgical procedures (PE) Cardiorespiratory investigations (DP) Patient thought it ‘ridiculous’ nothing wrong with heart or lungs (DR) ADLs, walking in apartment, talking, ability to leave apartment all affected (QOL)
Ok, Park and Id (2018)^88^	Quantitative	NQ	‘Healthy’ volunteers recruited from college students.	Korea	No funding received.	Lower mental and physical health related QOL (QOL)
Ostroglazov (1998)^42^	Quantitative	Clinical assessment	Referred to ‘local poly clinic’	Russia	Not disclosed.	Cardiorespiratory, psychological (S) Depression, phobias, fear of stopping breathing, neurotic syndromes, paranoid-hypochondriac, claustrophobia (PS)
O’Sullivan et al. (1992)^43^	Quantitative	HVPT with capnography	Referred to psychiatry clinic.	UK	Not disclosed.	Neurological (S) Physical or psychological trauma, demotion at work (PE) Cardiorespiratory and neurological investigations. BPD misdiagnosed as epilepsy (DP) ‘Tendency to present to hospital physicians’ (HU) Anxiety, depression, panic, phobias, agoraphobia (PS)
Peper et al. (2015)^71^	Case report(s), discussion paper	Not specified	Not specified.	USA	Not disclosed.	Neurological, psychological (S) Abdominal surgery 6-12 months previous (PE) Panic, anxiety, insomnia (PS)
Perkin and Joseph (1986)^55^	Quantitative	Not specified	Referred to neurology clinic.	UK	Not disclosed.	Neurological, cardiorespiratory, visual, gastrointestinal, other (S) Marital, domestic or financial problems (PE) Neurological investigations. BPD misdiagnosed as migraine, epilepsy or functional. Various other neurological conditions considered (DP) Two hospital admissions (HU)
Pincus (1978)^94^	Quantitative	HVPT	Referred to neurology clinic.	USA	Not disclosed.	Cardiorespiratory, neurological, ENT, gastrointestinal (S) More likely to have psychosomatic illness than controls (PS)
Richter (2021)^76^	Case report(s), conference abstract	Based on symptoms.	Not specified.	Not disclosed	Not disclosed.	Cardiorespiratory, neurological, other (S) Cardiorespiratory investigations (DP) A & E attendance (HU) Anxiety about health- feared contracted life threatening COVID-19 (PS) Bed ridden (QOL)
Roberts (1988)^56^	Case report(s), discussion paper	HVPT, ABG	Not specified.	USA	Not disclosed.	Cardiorespiratory, neurological (S) Father died of heart disease (PE) Told nothing was wrong, ‘nerves,’ need to relax (DP) Three A & E admissions in one month (HU) Anxiety about health- fears heart disease as father died of heart disease (PS) Comes home from work due to symptoms, drinking alcohol to ease symptoms during attack, unable to engage in usual activities as fears having a ‘spell’ (QOL)
Rogers (2019)^89^	Quantitative, conference abstract	CPET	Not specified.	UK	Not disclosed.	Participants with BPD have poorer QOL compared to the ‘normal’ population; mental, physical, role and social functioning affected (QOL)
Saccomani et al. (2014)^83^	Quantitative, conference abstract	Optoelectrical plethysmography	Not specified. Asthma diagnosis.	Not disclosed	Not disclosed.	BPD associated with anxiety in those with asthma (PS)
Saisch, Wessely and Gardner (1996)^59^	Quantitative	Capnography at rest, on exercise and voluntary over breathing.	Attended an accident and emergency department.	Not disclosed	Not disclosed.	Cardiorespiratory, neurological, psychological (S) Symptoms occur at rest or are stress induced (ET) History of abuse. Alcohol or drug misuse (PE) Cardiorespiratory investigations (DP) Multiple A & E attendees (HU) Anxiety about health- fears cardiac or other life threatening condition (PS)
Sedeh et al. (2017)^91^	Quantitative, conference abstract	NQ	Not specified. Potential severe asthma according to ERS/ATS 2014 guidelines.	Not disclosed	Not disclosed.	Lower QOL and poorer asthma control compared with severe asthma alone (QOL)
Sedeh et al. (2020)^92^	Quantitative	NQ, BPAT	Referred to respiratory clinic. Diagnosed with ‘difficult’ asthma.	Demark	The Danish Lung Association. Novartis Healthcare, Denmark.	Lower QOL and poorer asthma control compared with severe asthma alone (QOL)
Shu et al. (2007)^80^	Quantitative	ABG	Young males in military training presenting to accident and emergency with BPD.	Taiwan	No funding received.	A & E attendance (HU)
Smith (1985)^67^	Case report(s), discussion paper	Not specified	Not specified.	USA	Not disclosed.	Cardiorespiratory, neurological, gastrointestinal, other (S) Usually around mid-day (ET) Patient not accepting of diagnosis, wanted neurology clinic referral (DR)
Takeda et al (2024)^85^	Quantitative	NQ	From various hospitals and clinics. Asthma diagnosis.	Japan	No funding received.	Higher depression than asthma alone (PS) Poorer asthma control than asthma alone (QOL)
Van Dixhoorn and Duivenvoorden (1986)^97^	Quantitative	Clinical assessment	Referred for breathing and relaxation therapy. Clinic speciality not specified. BPD diagnosis.	Netherlands	Not disclosed.	Cardiorespiratory, ENT, other (S)
Wheatley (1975)^60^	Quantitative and case reports	Two step exercise test with double workload.	Not specified.	Not disclosed	Not disclosed.	Cardiorespiratory, neurological, MSK, other (S) Symptoms occurred at work when with dissatisfied customers. Walking down a certain hill but could do 18 chin ups. Sex. Heavy physical work. Walking fast for 5 miles. In bath or swimming in a lake. Strong emotions (ET) Supressing response to hypercritical mother (PE) Cardiorespiratory investigations (DP) BPD misdiagnosed as cardiac disease. Participant tearful and angry at clinician who gave wrong diagnosis (DR)
Wilson, Harley and Steels (2020)^77^	Retrospective audit	Clinical assessment	Transferred by regional ambulance service to two neighbouring UK hospitals.	UK	Not disclosed.	Cardiorespiratory, neurological, psychological, visual (S) Cardiorespiratory investigations (DP) Repeated A & E attendance, hospital admission (HU) Anxiety, mental health problems (PS)
Zraik et al. (2015)^72^	Quantitative, conference abstract	NQ, HVPT, capnography, CPET	Recent cardiac surgery.	France	Not disclosed.	BPD post cardiac surgery (PE) Exercise limitation (QOL)

*S* Symptoms, *ET* Episodic triggers, *PE* Precipitating experience, *DP* Diagnostic pathway, *DR* Diagnostic reaction, *HU* Healthcare use, *PS* Psychological factors, *QOL* Quality of life

*A & E* Accident and emergency department, *ABG* Arterial blood gas analysis, *ADL* Activities of daily living, *BPAT* Breathing pattern assessment tool, *BPD* Breathing pattern disorder*,* *COPD* Chronic obstructive pulmonary disease, *COVID* Coronavirus disease, *CPET* Cardiopulmonary exercise testing, *ENT* Ear, nose and throat, *ERS/ATS* European Respiratory Society / American Thoracic Society, *EtCO*_*2*_ End tidal carbon dioxide, *GERD* Gastroesophageal reflux disease, *HCP* Healthcare professional, *HR* Heart rate, *HVPT* Hyperventilation provocation test, *ICU* Intensive care unit, *ILO* Induced laryngeal obstruction, *NAD* Nothing abnormal detected, *NQ* Nijmegen questionnaire, *MSK* Musculoskeletal, *QOL* Quality of life, *RR* Respiratory rate, *RTA* Road traffic accident, *TcPCO*_*2*_ Transcutaneous carbon dioxide monitoring, *USA* United States of America, *UK* United Kingdom, *V*_*E*_ Minute ventilation

**Fig. 2:**
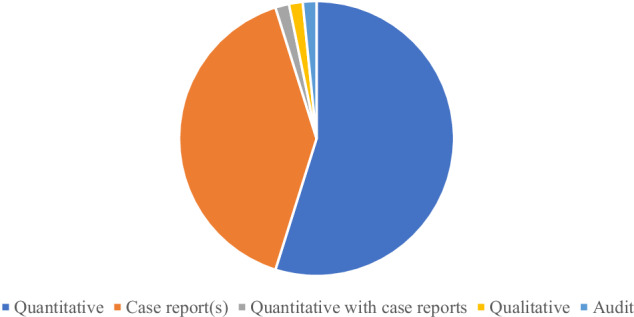
Report type.

The final 62 reports included in the review consisted of 34 quantitative reports, eight of which were published as conference abstracts. Twenty five case report(s) discussion papers, two of which were published as letters to the editor and two as conference abstracts. Additionally, there was one quantitative report which also included case reports, one qualitative report and one retrospective audit. Details of report type can be found in Fig. 2.

**Fig. 3:**
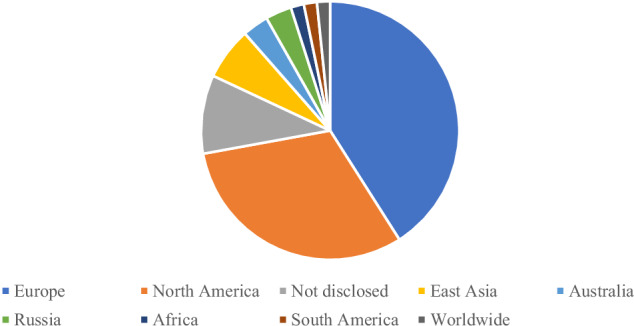
Geographical area of recruitment.

Twenty-five of the 62 included reports recruited from Europe, with 13 of these from the United Kingdom (UK). A further 19 reports recruited from North America, all of which were from the United States of America (USA). The rest of the world was poorly represented. One report recruited worldwide via the internet. Seven reports did not disclose their recruitment country. Details of geographical area of recruitment can be found in Fig. 3.

**Fig. 4:**
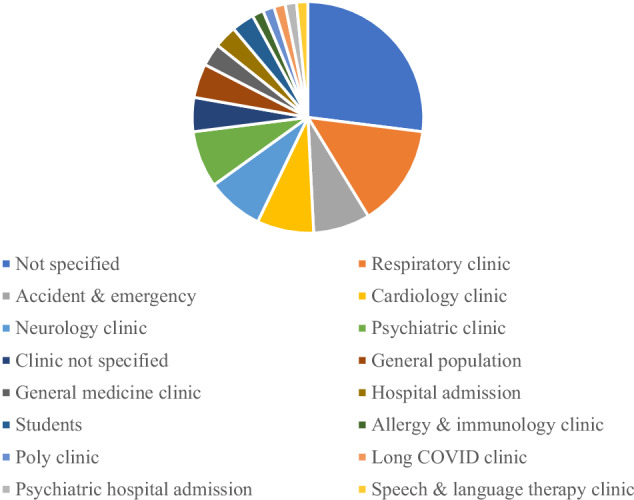
Participant recruitment population.

Participant recruitment source was very broad in terms of clinical speciality, as shown in Fig. 4. Thirty-three reports recruited from specialist clinics, with participants also recruited from accident and emergency, hospital wards including psychiatric hospitals, as well as general and student populations. Seven reports did not disclose their recruitment population and one report^35^ recruited from two different populations.

**Fig. 5:**
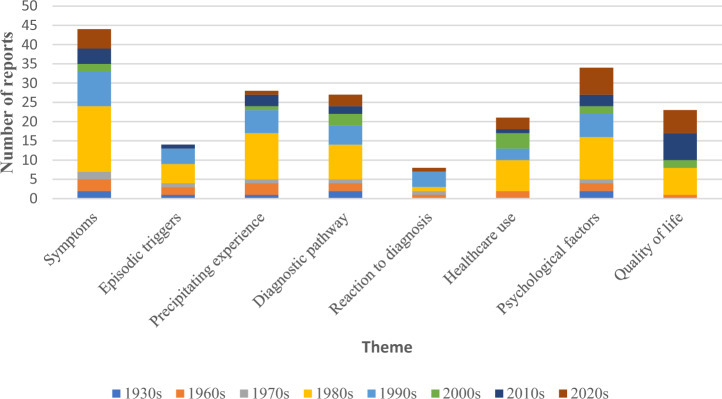
Number of reports published per decade for each theme.

The majority of data originated from the 1980s and 1990s for all themes except quality of life, for which over half of the reports came from 2010 onwards, although reports from the 1980s were still strongly represented within this theme. The ‘symptoms’ theme had the most evidence, with the least evidence found for ‘episodic triggers’ and ‘reaction to diagnosis’. A summary of the number of reports published per decade for each theme can be found in Fig. 5.

## Description of themes

The following themes emerged from the literature with regards to the lived experience of BPD; symptoms, episodic symptom triggers, precipitating life experience, experience of the diagnostic pathway, reaction to diagnosis, healthcare use, psychological factors, and quality of life.

### Symptoms

Patient reported symptoms of BPD were complex, diverse, and reflected a wide variety of body systems, often mimicking serious pathology. Table 4 presents BPD symptoms divided into body systems to demonstrate the wide reach of BPD and its ability to mimic other conditions.

**Table 4:** Patient and participant reported symptoms of BPD.

Body system	Symptoms
Cardiorespiratory	Breathlessness. Faster and/or deeper breathing A sense of being unable to take a deep satisfying breath, suffocation, smothering, air hunger or air ‘cutting off’, needing to breathe more. Chest pain, sometimes radiating into the arm. Palpitations, sensation of fast heart beat or ‘skipping beats.’ Excessive sighing, yawning, gasping. Dry cough.
Neurology	Bilateral or unilateral paresthesia, in limbs or side of face. Tetany. Dizziness, light-headed, giddy, vertigo, floaty sensation. Disconnected feeling, unreality, depersonalisation. Inability to concentrate. Headache, migraine. Trembling, tremor, twitching. Pseudo-seizures, absent spells, syncope, presyncope. Slurred speech, word forming difficulty
Musculoskeletal	Tension, pressure or tight sensation around the stomach and/or chest. Permanent soreness in mid-rift. Stabbing chest +/- arm pain. Tenderness in the chest or tender to palpation. Discomfort in and around chest. Stiff arms, hands, and/or feet. Tightness or stiffness around the mouth or face. Face, jaw or neck pain. Tight band sensation around head.
Ear, nose and throat	Muffled, hoarse voice, loss of voice. Sore throat. Dry mouth or throat. Swallowing difficulty. A sense of choking or globus Ear ache. Tinnitus.
Gastrointestinal	Abdominal pain or cramps Bloating, distension, gassy stomach, belching. Indigestion. Diarrhoea. Nausea, vomiting.
Psychological	Panic, fear, anxiety. A sense of impending doom, unease, unrest. Difficulty breathing triggered by stress or conflicts.
Visual	Blurred vision, loss of vision, photophobia, teichopsia.
Other	Fatigue. Hot or cold flushing sensations. Sweating.

References for Table 4^28,35–56,58–64,66,67,69–71,74–78,93,94,97^:

Each person appeared to experience their own, often unique, set of episodic symptoms. Episodes lasted from 45 seconds to over two hours^36,37^, with frequency of episodes occurring from one to two times a month to daily^38^, sometimes with a fluctuating pattern^39^. Duration of symptoms ranged from a few months to 14 years^38,40–43^. with symptoms sometimes occurring in childhood and then flaring up later in life^44^. Symptoms could be episodic at rest and poorly correlate with exertion, underlying cardiorespiratory pathology or physical findings^39,45–47^. Notably, a sense of being unable to take a satisfying breath was particularly common^36,38,39,45,46,48–56^.

### Episodic symptom triggers

Stress was a strong theme for episodic symptom triggers^23,52,57^, including mental stress^37,48,58–60^, conflict^48,58,61^, and strong emotions^60,62^, with Gilbert (1998)^63^ noting that even a brief mention of an upsetting experience may trigger episodic symptoms. Public gatherings, noise, hustle and bustle could also bring on symptoms^58,64,65^. Additionally, factor analysis by Bonde et al.^48^^, p.179^ identified that ‘Breathing difficulty triggered by mental stress/conflicts’ was a discriminating symptom of BPD. Physical activity, exercise, laughing or prolonged talking could also trigger symptoms^58,60,62^, however symptoms could also occur at rest for no reason^39,46,58,59,66^.

An inconsistent symptom pattern and context specific triggers appeared to be a feature of some BPD presentations, for example BPD symptoms would only occur at work^38,44,60^ with an absence of symptoms when on holiday^60^. While another gentleman usually only experienced his symptoms around mid-day^67^. Symptom triggers could also be activity specific^44,46,60,67^, for example one gentleman with BPD reported being able to do thirty chin-ups without chest pain, and yet experienced chest pain consistently when walking up a particular hill^60^. Another lady could square dance for hours, but experience breathlessness at rest^46^.

### Precipitating experience

The literature reported significant life events prior to BPD onset, as well as significant ongoing life experience. Bereavement appeared to be a very common trigger for BPD onset, especially the death of a close family member or friend, including still birth^39,43,47,49,58,61–63,68^. Work related stress, including fear of, or actual redundancy, were also common^36–38,43,44,54,62,64^, as were financial worries^37,55,58,69^ and relationship or family conflict^37,39,40,44,55,58,61,64,69^.

Other traumatic precipitating experience included childhood^50^ or domestic abuse^44^, physical^43,54,66^ or sexual^43^ assault, witnessing or being involved in a road traffic accident^58,61^ discovering a dead body^62,70^, murder of a family member^58^ or close friend^61^, or witnessing a suicide^62^.

Being a carer for a family member, especially at end of life^44,58^, loss of social connection with loved ones^39,54^, long-term resentment^39^, suppression of emotions^44,60^ or a sense of inadequacy or failure^44^ were also highlighted as precipitating experience.

Health related trauma such as surgery^39,45,47,58,62,71,72^, including a traumatic anaesthetic experience^61,62,70^, acute illness^45,47,50,73^ and resuscitation^62,70^ were also reported.

### Experience of the diagnostic pathway

The pathway to diagnosis of BPD could be prolonged and complex, which may affect the patient’s reaction to the diagnosis, once received. The diverse nature of symptoms, combined with no gold standard diagnostic method for BPD^14–17^, the need to rule out serious pathology^36,37^, necessitating multiple investigations and high healthcare use, could sometimes lead BPD to be diagnosed by process of elimination when no organic cause was found^40,45,46,58,74^. The literature reported a multitude of investigations, most commonly cardiorespiratory^41,43,45–47,54,58–60,62,64,66,69,75–77^, neurological^41,43,45,46,54,58,66,75^, or gastrointestinal focused^41,43,45,46,54,58,66,75^. Healthcare professional’s response to lack of organic findings could sometimes be negative with patients being accused of malingering^36,46^, hypochondria^56,78^ or suffering with nerves^56^. Lack of awareness of BPD by healthcare professionals, as well as patients reporting not being listened to, may also contribute to delayed diagnosis^78^. Understandably, this creates a prolonged diagnostic pathway that was often confusing and frustrating for both the patient and the clinician. Sometimes the wrong diagnosis was given^58^, with reports of treatment being started for conditions such as asthma^57,69,78^, epilepsy^38,43,55^ or cardiac disease^41,60^, for this diagnosis and treatment to be removed, and a diagnosis of BPD given, leading to patient stress, mistrust and uncertainty^38,41,43,55,57,58,60,69,78^. One study reported the mean duration of being incorrectly diagnosed with asthma was 7 years^57^.

### Reaction to diagnosis

Patient reaction to the diagnosis of BPD varied, however it was also poorly represented in the literature with the majority of insight coming from Byrne, Pfeffer and De Simoni (2023)^78^. Investigations showing nothing abnormal could be contentious, with some patients reporting feeling criticised, frustrated or feeling that they were a fraud^78^. Others rejected the diagnosis of BPD, believing that it was ridiculous that no abnormality had been found^47^, while others sought further investigations for organic causes^38,67^ or resisted acknowledging psychological factors^37^. Equally some patients were sceptical at first, only accepting the diagnosis after obtaining benefit from breathing retraining^78^. Other patients welcomed the diagnosis^78^, some were relieved that it was not a chronic life threatening condition such as asthma^69^, with one patient tearful and angry that she had been previously been wrongly diagnosed with cardiac disease^60^.

Clinician’s attitudes to BPD could also be negative. A clinician was reported to laugh at the idea that someone could ‘forget’ how to breathe, while other patients felt not listened to and unsupported^78^.

Explaining the concept of BPD could be challenging and elicit varied patient reactions. Negative patient reactions included a sense of being blamed and that symptoms were ‘habit’, or ‘a perceived feeling’ with ‘no actual cause’, ‘it’s just the way you breathe, or you aren’t trying hard enough.’^78^^, p.7^ However, one patient accepted the diagnosis following a physically focused explanation where ‘your body breathes quickly and shallowly without realising it…, but once it becomes normal for you your body isn’t able to do things that require good breathing, like exercise.’^78^^, p.8^ Furthermore, Howell (1990)^39^ found that patients more readily accepted diagnosis if present as physical condition, rather than linked to anxiety.

### Healthcare use

The symptoms of BPD were diverse and often mimicked serious pathology^37,41,59^ which may cause those with the condition to seek medical support. It appeared that the majority of healthcare use occurred as part of a prolonged diagnostic workup, or prior to diagnosis due to worrying symptoms without a diagnosed cause.

Concerning cardiorespiratory or neurological symptoms could lead to accident and emergency presentations^41,44,46,56,57,59,61,66,76–80^, sometimes frequent^41,46,56,79^. Saisch, Wessely and Gardner (1996)^59^ found 87% of those attending a UK accident and emergency department with symptoms of hyperventilation feared a cardiac or life-threatening condition, while a case study reported attendance of accident and emergency three times in ten days due to symptoms^79^.

Hospital stays were also reported, again with initiating symptoms usually cardiorespiratory or neurological^35,37,40,46,49,54,55,61,66,69,75,77,78^, sometimes with repeated admissions^40,46,54,55,77^. One patient, with BPD that was misdiagnosed as asthma, was admitted to intensive care^69^. Symptoms could even lead to psychiatric hospital admission^35,61^.

A retrospective audit by Wilson, Harley and Steels (2020)^77^ reported 3.05% of those with BPD (hyperventilation) taken by emergency ambulance to accident and emergency were admitted to a ward, with a length of stay between 2–9 days, while another 18.29 - 26.09% were referred to the onsite primary care centre. Of those diagnosed with BPD the re-attendance rate to accident and emergency within 28 days was between 19.51 - 26.09%, although the authors questioned whether this reflected high healthcare use or misdiagnosis, again highlighting the difficulty to accurately diagnose this condition.

### Psychological factors

The literature suggested a possible link between BPD and panic, anxiety, fear, phobias and depression, with the Hospital Anxiety and Depression Scale most frequently used as a measurement. Anxiety disorders, particularly panic and phobias were found to be a risk factor for BPD in those with asthma^81^. Anxiety was also shown to be associated with BPD without additional pathology^43,45,57,82^, as well as in those with a co-morbidity of asthma^83^ or COPD^84^. Panic disorder was also found to be associated with BPD^65,81^. Additionally, anxiety and panic were prevalent in case reports and an audit^35,39–41,44,61,64,71,77^. Anxiety regarding health, often fearing a serious or life threatening condition, or sudden death was common and understandable due to the nature of symptoms^28,35,39,54,56,58,59,61,68,76^. However, Koniukhovskaia (2022)^82^ noted an association between BPD and high and borderline levels of situational and personal anxiety during the COVID-19 pandemic, suggesting anxiety could be a precipitating factor for developing the condition.

Living in fear was also reported, including fear of being murdered^58^, a loved one dying^58^, the breakdown of a relationship^61^, rejection^62^, or redundancy^38,44^, as well as fearing an illicit relationship would be discovered^58^. Phobias were also commonly found in the literature^42,43,45,61^, with agoraphobia^43–45^, claustrophobia^42,62^ and fear of snakes^62,68^ most commonly reported.

Depression was prevalent in those with BPD^19,37,43,58^. When existing as a co-morbidity, people with BPD and asthma or COPD were found to be more depressed than those with asthma or COPD alone^57,84,85^. Depression was also noted in case reports^38,39,50,54,58,64^. Additionally, Maltinsky et al. (2025) ^86^, found that illness perception of people with BPD was negative, with the stronger belief that BPD was a serious condition, the more negative their mood and the greater the severity of anxiety and depression.

### Quality of life

BPD was found to have a negative impact on health related quality of life as measured by the Short Form 36 Health Survey Questionnaire across all^87^ or many domains^57,88,89^ with physical^87,89^, role^57,87,89^, and social function^57,89^, notably affected. Poorer asthma related quality of life, as measured by the Asthma Quality of Life Questionnaire, was also found in those with BPD and asthma, compared with asthma alone^90–92^. Asthma control, assessed via the Asthma Control Test^85^ or Asthma Control Questionnaire^90–92^, was also negatively affected by a comorbidity of BPD, with more frequent exacerbations also reported^81^.

Poorer COPD related quality of life was found in those with BPD and COPD, compared with COPD alone^84^. When BPD was a comorbidity to long COVID, health related quality of life was poorer, compare with long COVID alone^53,73^.

Participants with BPD believed the condition had a substantial impact on their life^86^. Poorer quality of life was also noted in case reports with the ability to leave the house^47,49^, carryout activities of daily living^41,46,47,56,64^, and participate in employment all negatively impacted by BPD^44,46,56,79^. Some reports described people being incapacitated^35^ or bedridden for days^76^.

## Discussion

The evidence for BPD experience is poorly represented in the literature. It is primarily viewed through a clinical lens from reports over 25 years old, with the majority of data originating from simple case reports, with empirical studies and an audit providing additional fragmented glimpses of experience. Only one study by Byrne, Pfeffer and De Simoni, (2023)^78^ provided direct patient voice, none of the other empirical studies focused on experience.

Authors were based, and participants recruited from, a broad variety of medical specialities, including psychiatry. This wide interest reflects the broad spectrum of BPD symptoms, spanning across many body systems, making the topic relevant to a wide variety of healthcare professionals.

Symptoms were complex and diverse, often mimicking serious pathology, which was understandably frightening for the patient, and could lead to high emergency healthcare use, especially when symptoms were unexplained prior to diagnosis. The prolonged diagnostic pathway, due to the need to rule out serious pathology, combined with the lack of agreed BPD definition or gold standard diagnostic tool, also contributed to high healthcare use and may promote diagnosis scepticism or rejection by some patients.

Psychological or physical stress were strong themes for episodic symptom triggers. Symptom triggers could be context or task specific, with symptoms poorly correlating with exertion or underlying pathology. Equally symptoms could occur at rest.

Precipitating experiences for BPD onset were also found to be stress related, often within the domains of work, financial or relationship, with ongoing stress possibly leading to chronicity of symptoms. Physical or psychological trauma, including abuse, ill health, surgery or bereavement were also common precipitating experiences.

Panic, anxiety, fear, phobias, depression and anxiety about health were often linked to BPD. However, it was unclear whether these were contributary factors in developing BPD, or were prevalent as a result of living with the symptoms of BPD. Additionally, panic, general anxiety, fear and anxiety about health may perpetuate symptoms, particularly within hyperventilation subtype of the condition.

BPD was associated with poor physical, social and role functioning, as well as reduced health and asthma related quality of life, and poorer asthma control.

### Gaps in knowledge

Further research exploring symptoms, episodic triggers and precipitating experience within the context of our current, broader understand BPD would be valuable in assisting earlier recognition of the condition. Such work may also contribute to our understanding of BPD aetiology, which remains poorly understood, as well as help identify those at risk of developing the condition.

Equally, further work is required to reach a consensus on BPD terminology, definition and diagnostic methods. Such work has the potential to expedite and improve the accuracy of the diagnostic process, which in turn could improve patient experience, reduce anxiety about health and possibly reduce healthcare use. Exploratory work focusing on how patients make sense of their symptoms and the diagnostic process, as well as understanding the differing patient reaction to the diagnosis would be valuable to inform how the diagnosis is presented to patients, which in turn may promote diagnosis acceptance.

The causal relationship between psychological factors and BPD remains unclear. Further research exploring whether these are precipitating factors, or common co-morbidities requiring treatment, would be valuable in guiding management.

Further work exploring the impact of BPD on quality of life may inform how best to support people living with the condition. Additionally, further research exploring BPD as a treatable trait of asthma, including exploring the validity of BPD diagnostic methods in those with asthma and how BPD affects asthma control and asthma related quality of life would be equally valuable.

## Limitations

The majority of reports included were over 25 years old and may therefore not reflect current experience, particularly diagnostic pathway experience, as diagnostic methods have progressed and developed over time.

Some of the reports recruited participants from accident and emergency^59,66,77,80^ where hyperventilation may have been caused by a one-off episode of anxiety or panic due to acute physical or psychological trauma, rather than chronic BPD.

The majority of the data comes from the 1990s or older when the hyperventilation subtype of BPD was the only form recognised at the time, until the early 2000’s when Thomas et al. (2001)^29^, used the broader term of ‘dysfunctional breathing.’ This may skew the findings to reflect the hyperventilation subtype of BPD. This change in terminology over time also complicated database searching, although a wide variety of search terms for BPD, and the ‘wildcard’ function allowing for various endings, was used to try to capture all relevant reports.

The change in BPD terminology and definition over time was also reflected in the diagnostic methods used in the literature, with these factors remaining unclear to the present day^17^. The hyperventilation provocation test (HVPT)^28,58^, capnography^40^ and later the popular NQ^18^, focused on hyperventilation, until the advent of broader assessment tools such as the MARM^20^, BPAT^19^, and CPET^15,23,24^, reflecting the changing understanding of BPD from purely hyper-ventilatory to a broader spectrum of dysfunctional breathing patterns^30^. This change in understanding, definition and diagnostic methods sheds doubt on the accuracy of diagnosis of BPD in the literature, particularly studies that used the NQ in isolation^73,79,82,85,88,90,91^ as it was not designed to be a standalone diagnostic tool^25^. Equally, the diagnostic accuracy of studies which relied solely on the HVPT, with or without capnography^28,35–39,41,43–46,54,58,61,62,68,70,93,94^, are also called into question as the specificity and validity of the HVPT has since been discredited^95,96^. Additionally, the majority of the data came from case reports where diagnostic certainty may be less robust than in empirical studies.

The vast majority of evidence came from the Western population and viewpoint, with almost three quarters of reports originating from Europe and North America, particularly the UK and USA. However, only studies published in English were included which introduced a language bias which may have excluded reports from diverse cultural contexts and therefore shaped how the experience of BPD is presented in this review. The included reports did not undergo critical appraisal, therefore the quality of evidence cannot be ascertained.

## Conclusion

The experience of living with BPD including symptoms, episodic triggers, precipitating experience, diagnostic experience, healthcare use, psychological factors and quality of life is poorly represented and predominately found Western based literature, with evidence mainly found in case reports over 25 years old, with snippets of insight found within empirical studies and an audit. This limited breadth and heterogeneity of the available evidence calls for further research in this field which employs more diverse and rigorous study designs to explore the lived experience of BPD, including the experience of the condition outside of Europe and North America.

## Supplementary information

Moffat BPD data extraction table v4.040226 clean

Moffat BPD database search strategy v1.0 101125

## Data Availability

The completed data extraction table for the themes has been provided as supplementary material.

## Materials availability

The database search strategy, including search terms and limiters, have been provided as supplementary material.
