# Supplementary material for: Living with breathing pattern disorder: a scoping review
Source: NPJ Prim Care Respir Med. 2026 Mar 25;36:30. doi: 10.1038/s41533-026-00495-5 (PMC13181008; doi:10.1038/s41533-026-00495-5)
Supplement: Supplementary file 1 — Moffat BPD data extraction table v4.040226 clean [file 41533_2026_495_MOESM1_ESM.docx]

**Living with breathing pattern disorder: A scoping review
Catherine Moffat; Susan Walker; Jonathan Fuld; Shanlee Higgins**

**Data extraction table**

| **Reference** | **Symptoms** | **Episodic triggers** | **Precipitating experiences** | **Diagnostic experience, including misdiagnosis** | **Diagnostic reaction: participant and HCP** | **Healthcare use** | **Psychological**  **factors** | **Quality of life** |
| --- | --- | --- | --- | --- | --- | --- | --- | --- |
| **Agache et al.,**  **2012**^1^ |  |  |  |  |  |  | Significant risk factor for BPD in asthma is anxiety, (HADS). Disorders, especially panic disorders and specific phobias of blood, injections and injuries. | Those with BPD and asthma were associated with the “frequent exacerbator phenotype” defined as at least 3 severe exacerbations requiring systemic steroids or hospitalisation in last 12 months. Also, a significant increase in severe and brittle asthma in BPD group compared to asthma alone. BPD also associated with GERD and rhinitis. |
| **Bass and Gardner**  **1985**^2^ | Facial pain, chest pain, palpitations, SOB, inability to take a satisfying breath, air hunger, panting, sighing, tetany, vertigo.  SOB disproportionate to physical findings. |  | Pacemaker fitted 6/12 plus possible PE previous to diagnosis. | “Extensive clinical investigations were usually performed before referred” to clinic.  Investigations showed no signs of any “obvious underlying organic condition.”  LFT, histamine challenge, V/Q scan, skin test for common allergens, CT brain, exercise cardiac stress test, coronary angiogram, fibreoptic bronchoscopy, ECG, echo, barium meal, gastroscopy, EEG, oral cholecystography.  Six cases “exhaustive investigations NAD.” |  |  | Anxiety, panic (crowds or spontaneous), agoraphobia, phobias. |  |
| **Blau, Wiles and Solomon**  **1983**^3^ | Unilateral arm weakness, unilateral facial weakness, Unilateral parathesis arm, Unilateral parathesis face, Numb arm, Unilateral facial parathesis, clumsiness of hand, Unilateral parathesis leg, cold feeling down one side of body, Numb leg, unilateral heaviness in chest, Heavy arm and leg, dizziness, tight chest, tension headache, dizziness, nausea, heavy chest, palpitations, panic attacks, blurred vision, fatigue, light headed, difficulty taking deep breath, breathlessness**.**  Episodes lasted a few mins. |  | Unable to find work. Anxious about job. | Investigated for epilepsy- negative, suspected cerebral vascular disease.  Previous diagnoses included epilepsy, TIA, demyelination, migraine.  Note: Table reports previous diagnosis of epilepsy, migraine, AVM, MS, CVD, brachial neuritis, angina, malingering, cerebrovascular insufficiency, ?these diagnoses co-morbidities or disproved- not clear.  Accused of malingering by HCP. |  |  |  |  |
| **Bonde et al.,**  **2013**^4^ | Difficulty in getting air in, difficulty in taking deep breaths, tenderness in the chest. | Breathing difficulty triggered by mental stress/conflicts. |  |  |  |  |  |  |
| **Brashear**  **1984**^5^ | Inability to get enough air, fighting for breath, staggering feeling, numb scalp, head, hands, blurred vision. |  | Bereavement. |  |  | Hospitalisation. |  | unable to leave apartment to socialise. |
| **Brodtkorb et al., 1990**^6^ | Numbness or paraesthesia with unilateral predominance, 5 left, 2 right, 1 left UL, right LL, rest bilat (N=25), dizziness, nuchal (back of neck) tension, headache, numbness in extremities, globus in throat, lightning stars and crescents moving in ascending manner in visual field (teichopsia), anxiety.  Episodic symptoms, episodes 5 – 30 mins. | Blind woman who looked after 1 year old child, symptoms would come on when child moved about. | 21/25 of participants had a history of a rather serious personal conflict or a complicated life situation involving work, economic matters or love. | Referral with suspected diagnosis to neurology clinic of epilepsy, TIA, MS, intracranial tumour, migraine.  Investigations- EEG. | “Our pts were concerned about somatic symptoms… but seemed to lack insight into psychological basis of their problems” | “A large fraction of pts were hospitalised with suspicion of fits of an epileptic nature” “pseudo seizures” | Minnesota Multiphasic Personality Inventory “More elevated scores” for hypochondria, depression, hysteria, paranoia, psychasthenia and schizophrenia.  Significant for hypochondria, depression, hysteria and psychasthenia. |  |
| **Byrne, Pfeffer and De Simoni**  **2023**^7^ | Hyperventilation, gasping, excessive yawning. |  |  | BPD misdiagnosed as asthma.  “Being told you’re a hypochondriac.” | Sense of being blamed and that symptoms were “habit”, or “a perceived feeling” with “no actual cause”, “it’s just the way you breathe, or you aren’t trying hard enough.”  “…I was diagnosed (with BPD) by a respiratory specialist, but a lot of healthcare professionals still laugh and ask me how I can forget to breathe”  “Its really difficult not knowing where you stand and being told you’re a hypochondriac, that’s it’s just the way you breathe, or you aren’t trying hard enough, and you don’t know what’s happening or how to make it better, especially if you are usually health and active”  “…its essentially where your body breathes quickly and shallowly without realising it, and sometimes you can hold your breath subconsciously too. It sounds insane, but once it becomes normal for you your body isn’t able to do things that require good breathing, like exercise. Physiotherapy can really help though. They can help you learn to breathe normally, and give you exercises to practice as well”.  “I was sceptical, but my physiotherapist persuaded me to try breathing exercises and they really helped, although I find them hard work as I have got a lot of bad habits …. I wish I had started them years ago”  Feared they were not believed, felt like a fraud and felt they had to ‘prove’ something was wrong when investigations for serious pathology came back normal.  Summary of main findings: Authors report most felt positive about formal diagnosis of BPD or ILO.  Suggestions BPD could be cause of symptoms could be met by frustration and perceived as suggestion of criticism. Others were positive and welcomed diagnosis.  Perceptions of HCP could be negative especially if felt unsupported or not listened to, partly due to lack of awareness of BPD and ILO meaning low rates of diagnosis and inappropriate management. | A & E visits and hospital admissions. |  |  |
| **Castro et al.,**  **2000**^8^ | Syncope, chest tightness, SOB, anxiety, presyncope. |  |  | Cardiac investigations, respiratory investigations, neurological investigations, blood tests, echo, coronary angiogram, tilt test, 24 hr tape, duplex doppler of ultrasound carotid and vertebrobasilar artery, MRI brain and brainstem, EEG, LFT, 12 hr pulse oximetry, ECG, ABG. |  | Hospital admission. |  |  |
| **Chenivesse et al., 2014**^9^ |  |  |  | LFTs, room air arterial blood gas, doppler echocardiogram, CPET, Nijmegen questionnaire, HVPT, Frensh Sadoul dyspnoea scale and Baseline Dyspnoea Index |  |  |  | Significant impairment in all health related QOL domains including physical (particularly physical role), mental and social dimensions, with a dramatic reduction in physical components. A dramatic reduction in physical functioning and role limitations due as a result of physical problems. “Much lower” SF-36 scores than other respiratory conditions cystic fibrosis, COPD or severe asthma, social anxiety or panic disorder. |
| **Cherif et al.,**  **2024**^10^ |  |  | 78.4% of participants had severe of critical forms of COVID-19 pneumonia.  Cognitive impairment (acute phase), persistent weight loss (post COVID phrase), PCFS grade>2 (post COVID 19 functional status score) and PTSD as independent factors for developing HVS. |  |  |  | PTSD as independent factors for developing HVS. |  |
| **Chevalier and Schwartzstien**  **2000**^11^ | SOB, need to breathe more, not getting a normal deep breath, smothering sensations, parathesis, dizziness, respiratory discomfort, breath does not go all the way in, hoarse voice, inability to get a full breath, suffocation. |  | Pneumonia. Childhood abuse. | Cardiac and respiratory investigations NAD. V/Q scan, CXR, echo, LFT, CPET, Thoracic CT, ABG. |  |  | Depression. |  |
| **Conway, Freeman and Nixon**  **1988**^12^ |  |  | Bereavement close family member. Rejection. Loss of physical control. Anger. |  |  |  | Fear of snakes. Anxiety about health. |  |
| **Decuyper et al.**  **2012**^13^ | Palpitations, SOB, nausea, dizziness, headache, muscular pain, fatigue, tinnitus, paraesthesia, blurred vision, anxiety, sweating, concentration problems, gastric complaints. |  |  | Referred to psychology dept by neuro, neuro surgeons, geriatrics, respiratory, cardiology, internist, rehabilitation, gastroenterology, ENT psychiatrists with no organic disease found and symptoms through to be hyperventilation. |  |  |  |  |
| **Denton, Bondarenko and Hew**  **2018**^14^ |  |  |  |  |  |  |  | Asthma control was poor at baseline with mean of 3.1 severe exacerbations in the 6 months prior to review.  BPD associated with poor asthma control and asthma related quality of life.  ACQ (mean score of 2.4) and AQLQ (mean score of 3.9). |
| **Evans**  **1995**^15^ | Near syncopal episodes, lighted headed, 1 min daily, 10 week history, 1 yr history, 1-2 times a month less than 1 min, feeling faint/ fading away with SOB, 5 yr history, last about 45 seconds, occurring every 2-3 days for last 3 months, strange feeling, feeling of confusion, déjà vu, coldness and numbness left side of body, brief LOC, past few weeks daily episodes of light headedness, nervousness, weak feeling and numbness left arm, left leg, perioral area, chest tightness, trouble getting a good breath, lasted “minutes”, 1 month history, lasted 2 mins or less, many times a day, light-headedness, numb left upper lip, left side head, feeling left arm as asleep, speech slightly slurred. | Usually sitting at work, in meetings at work. | Layoffs at work. | Previous ENT and internist clinic review, MRI brain, EEG, blood tests, exercise testing, MRI brain with angio sequence.  Partial seizures diagnosed- carbamazepine prescribed but then diagnosis removed and treatment stopped when BPD diagnosied. | Patient unconvinced by BPD diagnosis, wanted MRI to rule out other causes. |  | History bipolar with many psychiatric admissions. Increased depression same time as onset of symptoms. Feeling of smothering when in crowds.  Fear of redundancy. |  |
| **Freeman, Conway and Nixon 1986a**^16^  ***Published- Royal Society of medicine.* *Focus on ETCO_2_*** | Tightness in neck and left arm, blackouts. | Exercise.  Strong emotion. Anger at boss at work. | Work stress. Bereavement.  Loss of control from resuscitation for recurrent VF post MI (loss of control of body to doctors). Saw suicide. Discovered a dead body. General anaesthetic recovery room- repeatedly told to “come on breathe.” | Previous extensive investigations by respiratory clinic. |  |  | Fear of snakes, fear of enclosed spaces (claustrophobia).  Fear of rejection. |  |
| **Freeman, Conway and Nixon 1986b**^17^  ***Published- Journal of Psychosomatic Research. Focus on HR*** | Palpitations, blackouts, chest pain, neck pain. |  | Sense of loss of control during myocardial infarction with repeated resuscitation attempts. Discovery of a dead body. Anger at loss of self-control when aroused. |  |  |  |  |  |
| **Gardner and Bass 1989**^18^ | SOB, chest pain, cardiac neurosis, invalidism, tetany, paraesthesia. |  | Heart murmur as a child. |  |  | Admission to psychiatric hospital. | Panic attacks. Lifelong fear of cardiac disease following detection of a heart murmur as a child, anxiety about health. | Incapacitated for 2-3 days. |
| **Gilbert 1998**^19^ | Chest pain, light-headedness, neck and shoulder fatigue, SOB. | Brief mention of upsetting experience may trigger symptoms. | Trapped in an elevator with non-functioning intercom. Bereavement from death of family member. |  |  |  |  |  |
| **Goff and Gaensler**  **1969**^20^ | SOB, dizziness, faintness, right leg pain. |  | Intrafamily conflict. | Bloods, EEG, ECG, CXR, LFT, ABG-resting and post exercise, ETCO_2_, angiography. |  | 2 hospital admissions. | Anxiety. |  |
| **Greene 1984**^21^ | Hoarse voice, periods of aponia, difficulty forming words, globus, dizziness, headache, tingling fingers, dry throat and mouth, insomnia, permanent soreness in the midriff, day-dreaming, difficulty concentrating, fainting fits, dizziness, air hunger, palpitations, blurred vision, head and neck ache, stabling pain in chest, bruised feeling in arm, abdominal pains.  Three year history.  Fainting and falling only occurred at work, symptoms occurred in childhood but flared up later in life**.** | Presenting at business meetings.  Symptoms context specific. | Profound sense of failure and inadequacy.  Marital problems, unhappily situated in Germany as wife of a service man. Mum left home 2 years previous had been left with sister to run the home and look after bronchitic and alcoholic father- felt great responsibility to look after him esp after sister married, worked long hours at a shop where she was a manger. Physical and emotional domestic abuse (left fiancé who bullied and “knocked her about”). Critical father as a child. Hold up-right posture, appear brave even if fearful. | Laryngoscopy. |  | A & E attendance. | Anxiety, panic attacks, fear of flying, agoraphobia.  Fear of redundancy. | Fainting and falling at work. |
| **Grossman and De Swart 1984**^22^ | Faster or deeper breathing than normal, pounding heart, dizziness, fits of crying, feelings of unrest or panic, feeling anxious, trembling hands. |  |  |  |  |  |  |  |
| **Hagman, Janson and Emtner**  **2008**^23^ |  |  |  | Previously treated with asthma medication. Miss diagnosis of asthma. Had breathing problems without correct diagnosis for mean of 7 years. |  | Often made A & E visits due to breathing problems. | Significantly more anxious and depressed than those with asthma. Those with dysfunction breathing more impacted by stress than those with asthma | Health related QOL SF-36 significantly lower in vitality, social functioning and role emotion. Significantly more breathing problems than those with asthma alone. More negatively affected by stress and breathing problems had more impact on their daily lives. Poorer sense of coherence. |
| **Han et al., 1998**^24^ | Headache, blurred vision, SOB, unable to breathe deeply, discomfort in or around chest, faster deeper breathing, chest pain, dizziness, derealization, stiff fingers or arms, tightness around the mouth, tingling fingers, anxiety, tenseness, palpitations, tightness around the mouth, bloated abdomen, cold hands or feet. |  |  |  |  |  |  |  |
| **Hegel et al., 1989**^25^ | Four year history of chest pain, Chest pain- 1 year duration, upper chest heavy breathing, frequent gasps and sighs, trembling, dizziness, SOB, fainted. |  |  | CXR, echo, neurological exam, treadmill exercise test, GI endoscopy. Treated as angina. Treated as possible HF. |  | “Several” A & E.  3 A & E visits. | Panic attacks. | “Activity limited by chest pain.” |
| **Hoes et al., 1987**^26^ |  |  |  |  |  |  | Panic disorder significantly more prevalent in people with BPD. |  |
| **Howell 1990**^27^ | Symptoms poorly correlated with exertion, symptoms at rest, fluctuating and recurrent.  Harder to breathe in than out, dizziness, light headed, paraesthesia, cramps, hot and sweaty when SOB, SOBAR or SOBOE or both, palpitations, parathesia- sometimes unilateral, usually left. | Symptoms at rest for no reason, when relaxed.  Symptoms poorly correlated with exertion, symptoms at rest, fluctuating and recurrent. | Events of preceding three years sought in report:  Previous family history of depressive illness. Bereavement (close relative with severe and prolonged grieving) 8yr old son wandered on to railway and killed by train.  Separation (removal of a foster child or emigration of a son/daughter).  Current martial disharmony. Gross secondary gain from “illness.” Living alone. Previous surgery. Resentment about the way the patient or close relative/friend had been treated (failed to get a promotion, treated badly by doctor).  Failure to receive reassurance about an insignificant but worry symptom- when symptoms are unexplained fear of illness becomes dominant.  Dizziness, feared brain tumour- neurologist investigated- NAD, but no convincing explanation given for dizziness, when reassured nothing wrong may fear mental illness. |  | Patients more readily accepted BPD diagnosis if explained as a physical condition and avoid implying its “nerves” due to anxiety or tension. |  | Anxiety, depression, perfectionist traits.  Fear of sudden death during symptom episode. Anxiety about health.  Uncertain whether they have a serious illness.  Fear will die during an attack. |  |
| **Huey and West**  **1983**^28^ | Pressure or tight band sensation around head or neck, light-headedness, dizziness, faintness, SOB- can’t seem to get enough air. |  |  |  |  |  |  |  |
| **Kang 2015**^29^ | Altered consciousness, recurrent tremulous movements jaw and hands. |  | Physical assault. | ABG, blood analysis, urinalysis, ECG, head CT, EEG, head MRI. |  | A & E attendance, hospital admission. |  |  |
| **Kerr et al., 1937**^30^ | Convulsive seizures, generalised tonic colonic convulsions, -9 months, 6 weeks, 8 yrs, Spasms hands and feet (carpopedal spasm of tetany- 1 month, 1 year) cramps and feet, air hunger, stiff hands and feet, numbness hands and feet, difficulty swallowing due to stiff throat muscles, gaseous distension and diarrhoea, “puckering” sensation around mouth, parathesia chest wall, hands, feet, fatigue, irritability, nervousness, trembling, seating, flushing face, arms, chest, headache, stiff jaw, swollen eye lids, abdominal distension, hands and arms ache, general malaise, cough, SOB, chest pain, clenching fists, kicking feet, chest pain, chest pain radiating down right arm/ left arm, left scapular, nausea, blenching, photophobia, twitching of muscles in the body, rapid HR, palpitations (1 year), tension in chest, insomnia, frequent sighs, faintness, blanched fingers, air hunger, choking sensation throat, sensation of clutching compression throat, suffocation, tense feeling throat, epigastric pain, intolerance to heat, fullness in neck, tension in chest on inspiration, general weakness, neuromuscular twitching, sense of suffocation and tightening of facial muscles if laughed or talked too much, belching, vertigo. | Visits from mother-in-law. Excitement or argument. Altercations with family. Drinking alcohol at public gatherings. Emotional upsets and altercations. Distressed about finances or health. Laughing or talking too much. | Started divorce proceedings. Divorce. Husband drank a lot of alcohol. Death of husband. Death of ex-husband. Witnessed RTA. Injection for dental work. Father died from MI- she nursed him. Restrictions on social life due to skin disease (severe eczema). Bereavement- death of father, husband. Hand injury. Brother-in-law had killed his wife and wife’s family. Disliked father-in-law. Nursed mother through fatal attack of angina. Birth of child (mother). Financial worries.  Caring for paralysed husband she did not love. Marital disharmony.  Surgery- subtotal thyroidectomy for Graves’ disease. Other cases of sub-total thyroidectomy. Thyroidectomy. | Organic disease ruled out.  Pulse, BP, reflexes, Trousseau’s and Chvostek’s sign, urine analysis, blood analysis, stool analysis, EEG, Wassermann and Kolmer reactions negative, gastric analysis, ECG, symptoms simulated asthma, urine analysis, gall bladder scan. |  |  | Depression, insomnia.  Anxiety about health.  Clandestine romance with uncle, fears being found out.  Brother-in-law had killed his wife and wife’s family- feared her husband would do the same.  Fear of losing her husband due to her chronic illness. |  |
| **Kerr, Gliebe and Dalton**  **1938**^31^ | Abdominal discomfort, rapid HR, palpitations, constriction around throat, sense of suffocation, frequent sighing, gasping, stiffness hands and feet, dizziness, nausea, weakness, fatigue. |  |  | BPD wrongly diagnosed as a serious pathology. |  |  | Serious pathology wrongly diagnosed added to anxiety about health. |  |
| **King 1984**^32^ | Palpitations, “skipped beats” SOB, chest pain- ache, chest discomfort, flushing of the face, headache, light headedness, tingling both hands and feet particularly left side, constriction in throat, difficulty swallowing, | Noise and bustle. | Work problems. Marital problems. | Coronary artery disease suspected but Angiogram normal. Exercise test. |  |  | Anxiety, depression. | Unable to do most of her cooking, can’t do any housework. |
| **Koniukhovskaia et al.,**  **2022**^33^ |  |  |  |  |  |  | Association between BPD and high and borderline levels of situational and personal anxiety during the COVID 19 pandemic. |  |
| **Lapperre et al.,**  **2020**^34^ |  |  |  |  |  |  | Significantly higher anxiety and depression in COPD and BPD than COPD alone. | Poorer COPD related QOL than COPD alone. |
| **Lazarus and Kostan 1969**^35^ | Numbness and pins and needles arms and legs, dizziness, altered consciousness, stomach pains, headaches, SOB. | Driving in car being far from home or destination. Argument with husband. | Martial disharmony. Not able to morn mothers death. Husband flirting with another woman. RTA. Unconsciousness from anaesthesia- state of helplessness under medication. Close friend was shot and killed by her husband. |  |  | Hospitalisation by psychiatrist during acute episode.  A & E attendance. | Anxiety, fear of being alone, fear of the dark.  Anxiety about health. Feared heart disease, heart attack, or brain tumour. Feared she was losing her mind.  Fear of breakdown of relationship with husband. |  |
| **Loew et al.,**  **2022**^36^ | Most frequent NQ items- faster or deeper breathing, unable to breathe deeply, sighs, yawning, tight feelings in chest. |  |  |  |  |  |  | Those with BPD had SF-36 scores lower than population reference value. |
| **Magarian 1982**^37^ | Brief episodes of unconsciousness, light headedness, dizziness, feeling of unable to take full inspiration, palpitations, tingling and numbness around mouth and hands, numbness and tingling sensations (did not say where), bloating, fatigue, weakness, presyncope, SOB, fullness in throat, sense of impending doom, chest pain, unsteady feeling, panic, agitation, depersonalisation. |  | Friends, family members and husband “drifting away” felt extremely isolated. Stabbed in abdomen at work. Unemployed for extended period. Wife receiving psychiatric care. | Carotid and vertebral angiography, ECG. CCF considered but ruled out. |  | Several hospital admissions. | Nervousness, depression.  Feared he would die of symptoms. |  |
| **Magarian and Olney 1984**^38^ | Verbal non-responsiveness, retrograde amnesia minutes to hours in length, chest pain, SOB, dizziness, light headedness, unsteadiness, blurry vision, peripheral and perioral (around the mouth) numbness and tingling, muscle cramping, carpel pedal spasms, inability to take a satisfying breath, sighing, fatigue, depersonalisation, palpitations, chest pain. | SOB at rest but could square dance for hours without SOB or chest pain. |  | Diagnosed with unusual seizure disorder, brain tumour, stroke, cardiac arrhythmia or no disease at all.  Neurological investigations, cerebral angiogram, EEG, CT brain, lumbar puncture, treadmill stress test, ambulatory ECG,  Accused of malingering. |  | Multiple hospital admission, frequent A & E, multiple outpatient clinics. |  | Impaired function at home and work as security guard. |
| **Maltinsky et al., 2025**^39^ |  |  |  |  |  |  | Illness perception of BPD was negative. The stronger the believe that BPD was a serious condition the mood negative their mood Strong positive relationships between illness perceptions of those with BPD and psychological outcomes such as severity of anxiety and depression. | Individuals believed BPD was a chronic concerning condition with severe symptoms and substantial life impact. |
| **Mooney and Candy 2008**^40^ |  |  |  |  |  | A & E 3 occasions in 10 days. |  | 12 days sick leave- economic cost. |
| **Nguyen et al.,**  **1992**^41^ | SOB |  | Financial and martial difficulties. | Diagnosed and treated as asthma, inhalers prescribed.  LFT, ABG, CXR, bronchial provocation test. | Relieved on diagnosis of BPD that she did not have chronic life threatening disease such as asthma. | Admitted to ICU and treated for asthma. |  |  |
| **Noehren 1966**^42^ | SOB chest pain, sweating. | Inconsistent symptoms.  Emotional stress.  SOB with and without exertion. SOB on talking. Well on waking but then sudden SOB and sweating 20 mins after wake up but resolve spontaneously.  Visited friend in hospital for 1 hour- could climb many flights of stairs beforehand, but after visited friend was unable to climb 4 steps without severe SOB (effects of emotional visit). | Frequent bouts of pneumonia. Several unrelated surgical procedures, followed by post op “black outs.” Mother died of pneumonia 59yrs old, father died of heart disease 65yrs. Favourite uncle died at the onset of her pneumonia- when all her symptoms started.  Understanding precipitating factors became an important part of treatment. | CXR, ECG, blood and urine tests, ABG, lung function tests. | Pt thought it was “ridiculous” that there was nothing (acutely)wrong with her heart and lungs.  Required reassurance she could do exercise such as walking in corridor and upstairs. |  |  | “Combing her hair exhausted her.” “Just getting around apartment or maintaining a simple conversation had become almost impossible.”  Reduced ability to leave apartment to attend hairdressers- attending less- 3-4 times in 7 months. |
| **Ok, Park and Id 2018**^43^ |  |  |  |  |  |  |  | Significantly lower mental and physical health related QOL in BPD group. |
| **Ostroglazov**  **1998**^44^ | Had symptoms for 2-14 years before clinical assessment.  “Hardly noticeable unease and mild lack of air hunger arising together with the dis-automatization of involuntary breathing”, “serious attack of panic with suffocation and forced respiration resembling asthmaticus.” Sense of oxygen starvation, difficulties breathing, high respiratory rate from the chest, increased depth, irregular, deep breaths like yawning. A sense of dis-automatization of involuntary breathing as pt tries to compensate for sense of insufficient oxygen uptake by voluntarily increasing rate and depth. Sense of suffocation. “tormenting suffocation” “burdensome obstacles interfering with a freedom of respiratory movements.” |  |  |  |  |  | Phobias. Fear of enclosed or crowed spaces (claustrophobia). Fear of suffocation. Fear of stopping breathing if not focusing on voluntarily breathing.  Participants: 11/24 (46%) depression, 10/24 (42%) neurotic syndromes, 3/24 (12%) paranoid-hypochondriac states. Fear of death from suffocation, “obsessive fear of respiratory paralysis”. |  |
| **O'Sullivan et al., 1992**^45^ | Duration of symptoms 1-11 years.  7/9 left side affected, unilateral somatic symptoms, epileptic like seizures (5/9). |  | Still birth. Physical illness. Assault. Sexual trauma. Demotion at work. | EEG, ECG, lung function tests, head and thoracic CT, 2/9 diagnosed as epilepsy and prescribed anticonvulsants with little effect. |  | “Tendency to present to hospital physicians.” | Major depressive episode. Panic disorder with agoraphobia. Agoraphobia without panic disorder. Somatisation disorder. Generalised anxiety disorder. Conversion disorder. Panic without agoraphobia. Anxiety and depressive symptoms (5/9 of participants). |  |
| **Peper et al.**  **2015**^46^ | 12 psychogenic epileptic seizures a week. |  | Colon surgery 6 months ago. 1 year after appendectomy. 6 months after kidney transplant.  Upper chest breathing to avoid pain post-surgery. |  |  |  | Anxiety. Feared could not breathe. Panic. Insomnia. |  |
| **Perkin and Joseph 1986**^47^ | Giddiness (faintness, dizziness, light headedness, vertigo). Paraesthesia (numbness, parathesia- UL, LL, face, trunk, sometimes unilateral). Loss of consciousness. Visual disturbance (blurred vision, loss of vision, photophobia, flashing lights). Headache. Nausea, ataxia, tremulousness, tinnitus. SOB, inability to get enough air into the chest, palpitations, chest discomfort. Insomnia, tiredness, abdominal pain. |  | Marital, domestic or financial problems. | Full neurological exam, EEG.  Wrong diagnosis: epilepsy, complex partial seizures, previously, migraine.  One patient had received a trial of anti-convulsant medication.  Alternative diagnosis suggested by referral letters- epilepsy, vaso-vagal attacks, MS, functional, migraine, hypoglycaemia, brain tumour, vertebra-basilar insufficiency. |  | 2 hospital admission for seizures. |  |  |
| **Pincus 1978**^48^ | Percentage of participants showing the symptoms of: 86% 2 organ systems, 30% 3 or more organ systems, neurological (light headed) 80%, paraesthesia 50%, headache 37%, weakness 27%, inability to concentrate 23%, loss of consciousness 6%, tetanus 3%, inability to swallow 23%, abdominal pain 10%, SOB 23%, palpitations 20%. Chest pain 17%. |  |  |  |  |  | Those with BPD significantly more likely to have psychosomatic illness that controls. |  |
| **Richter 2021**^49^ | SOB, fatigue, dizziness. |  |  | Lung function tests, CT chest. |  | A & E attendance. | Despair.  Anxiety about health- feared contracted COVID 19, feared for life.  Fears he will pass out. | Bed ridden. |
| **Roberts 1988**^50^ | “Can’t get enough air” chest pain, numbness and tingling left arm, palpitations, inability to breathe. |  |  | Told nothing is wrong, and that they suffer with nerves, and need to relax. |  | A & E three times in last month. | Anxiety about health. Fears heart disease (father died young of heart disease). Fears becoming an alcoholic like his mother. | Unable to engage in usual activities as feared having a “spell.” Comes home from work due to symptoms. Drinking alcohol to ease symptoms during an attack. |
| **Rogers 2019**^51^ |  |  |  |  |  |  |  | Significantly poorer QOL compared to the normal population. Mental, physical, role and social functioning significantly aspects affected. |
| **Saccomani et al., 2014**^52^ |  |  |  |  |  |  | BPD significantly associated with anxiety in those with asthma. |  |
| **Saisch, Wessely and Gardner**  **1996**^53^ | SOB (61%), paraesthesia (35%), chest pain or tightness (43%), muscle spasm (9%), dizziness (13%), palpitations (13%) anxiety and panic (30%), previous similar episodes 74%.  Only 17% recognised they were hyperventilating. | Resting or stress induced. | Past abuse. Alcohol or marihuana use. Fever. Drug or alcohol abuse. | ABG, blood tests, CXR. |  | A & E attendance.  Most participants had previously attended A & E due to BPD. | Anxiety about health. Feared cardiac or other life threatening condition (CVA, lung cancer, hypoglycaemia).  Fear of serious disease (87% of participants) was the main reason for attending hospital. |  |
| **Sedeh et al.,**  **2017**^54^ |  |  |  |  |  |  |  | Significantly lower QOL and significantly poorer asthma control than difficult asthma alone. |
| **Sedeh et al.,**  **2020**^55^ |  |  |  |  |  |  |  | Significantly lower QOL and significantly poorer asthma control than difficult asthma alone. |
| **Shu et al., 2007**^56^ |  |  |  |  |  | A & E attendance with BPD. | Risk of BPD increased by greater neuroticism, less extraversion, parental overprotection, less parental caring.  (Note: psychoanalysis not included as a theme). |  |
| **Smith 1985**^57^ | Dizziness, fatigue, dreamy, floaty sensation, stiff arms, dry mouth, occasional paraesthesia one arm, unilateral facial numbness, abdominal cramping and diarrhoea. | Symptoms often occurred around mid-day but occasionally at other times as well. |  |  | Patient asked for neurologist referral when no organic cause was found for symptoms. Not accepting of BPD diagnosis. |  |  |  |
| **Takeda et al.,**  **2024**^58^ |  |  |  |  |  |  | Significantly higher depression than asthma alone. | Significantly poorer asthma control than asthma alone. |
| **Van Dixhoorn and Duivenvoorden**  **1986**^59^ | Tight sensation around stomach and ribs, forget to inhale, sigh a lot, speaking quickly- lots of words single breath, long time passes without breathing after exhaled, loss of voice. |  |  |  |  |  |  |  |
| **Wheatley 1975**^60^ | Chest pain, rapid deep breathing, wet palms, perioral and extremity paraesthesia, SOB, chest pain like elephant sitting on chest, dizziness, sense of suffocation, tenderness chest, twitching eye lids, like an orange sitting in my chest, pain radiate from chest to hands, felt weak, nausea. | At work selling auto parts dissatisfied customers made him feel like he was “going to hell”. When lift 60lb oil cases. Lunch hour rest prevented afternoon chest pain. A good night’s sleep prevented morning symptom “attacks” but symptoms would come back in the afternoon when more fatigued. Symptoms disappeared on vacation but returned when back at work. Chest pain disappeared after changing employment. Pushing cars through snow. Sat in bath. Climbing 88 steps to home. Chest pain during sex. Walking downhill to work yet 2 weeks later could walk rapidly up same hill with no symptoms. Secretly leaving work early would cause chest pains at checkout clock felt weird and arms felt funny yet could walk rapidly 5 miles to work and do 18 chin ups without pain. Lifting heavy garbage barrels. Husband lost both hands she often assumed the more strenuous role during sex. High emotions. | Coped with hypercritical mother by keeping mouth shut.  Secretly leaving work early. | Lung function tests, blood tests ECG rest and on exercise.  BPD wrongly diagnosis as atherosclerotic heart disease. | Assurance symptoms were BPD caused tearful hostility towards previous cardiologist who thought she had cardiac disease- anger at previous wrong diagnosis. |  |  |  |
| **Wilson, Harley and Steels**  **2020**^61^ | Fast respiratory rate, feeling anxious, chest pain, SOB, fear, paraesthesia limbs/face, dizziness, palpitations, feeling confused or unreal, blurred vision. |  |  | lung function tests, ECG, ABG, VBG, CXR, D-dimer. |  | A & E attendance. 3.05% taken by ambulance to A & E were admitted to a ward with length of stay between 2-9 days, while another 18.29 - 26.09% were referred to the onsite Primary Care Centre. The re-attendance rate within twenty eight days to accident and emergency of those diagnosed with BPD was between 19.51 - 26.09%, although the authors question whether this reflects high healthcare use or misdiagnosis, again highlighting the difficulty to accurately diagnose this condition. | Anxiety “mental health problems.” |  |
| **Zraik et al.,**  **2015**^62^ |  |  | BPD post cardiac surgery causing exercise limitation. |  |  |  |  | Exercise limitation. |

**Key**

24 hour tape: ECG Holter monitor

A & E: Accident and emergency

ABG: Arterial blood gas

ACQ: Asthma Control Questionnaire

AQLQ: Asthma Quality of Life Questionnaire.

AVM: Arterial venous malformation

BP: Blood pressure

BPD: Breathing pattern disorder

CCF: Congestive cardiac failure

COPD: Chronic obstructive pulmonary disease

COVID 19: Coronavirus disease 2019

CPET: Cardiopulmonary exercise testing

CT: Computed Tomography

CVD: Cerebral vascular disease

CXR: Chest x-ray

Echo: Echocardiogram

ECG: Electrocardiogram

EEG: Electroencephalogram

ENT: Ear, nose and throat

ETCO_2:_ End tidal carbon dioxide

GI: Gastrointestinal

HADS: Hospital anxiety and depression scale

HCP: Healthcare professional

ICU: Intensive care unit

ILO: Inducible laryngeal obstruction

LFT: Lung function tests

LL: Lower limb

MI: Myocardial infarction

MRI: Magnetic resonance imaging

MS: Multiple Sclerosis

NAD: Nothing abnormal detected

NQ: Nijmegen questionnaire

PCFS: Post COVID 19 functional status score.

PE: Pulmonary embolism

PTSD: Post traumatic stress disorder

QOL: Quality of life

RTA: Road traffic accident

SF-36: 36-item short form health survey

SOB: Shortness of breath

TIA: Transient ischaemic attack

UL: Upper limb

VBG: Venous blood gas

V/Q: Ventilation/perfusion

**References**

1. Agache, I., Ciobanu, C., Paul, G. & Rogozea, L. Dysfunctional breathing phenotype in adults with asthma - incidence and risk factors. *Clin Transl Allergy* 2, 1–7 (2012).

2. Bass, C. & Gardner, W. N. Respiratory and psychiatric abnormalities in chronic symptomatic hyperventilation. *Br Med J* 290, 1387–1390 (1985).

3. Blau, J. N., Wiles, C. M. & Solomon, F. S. Unilateral somatic symptoms due to hyperventilation. *Br Med J* 286, 1108 (1983).

4. Bonde, E., Andersson, E., Brisman, J., Ringsberg, K. C. & Torén, K. Dissociation of dysfunctional breathing and odour intolerance among adults in a general-population study. *Clin Respir J* 7, 176–182 (2013).

5. Brashear, R. E. Hyperventilation syndrome: managing elderly patients. *Geriatrics* 39, 114–125 (1984).

6. Brodtkorb, E. *et al.* Hyperventilation syndrome: clinical, ventilatory and personality characteristics as observed in neurological practice. *Acta Neurol Scand* 81, 307–313 (1990).

7. Byrne, C., Pfeffer, P. E. & De Simoni, A. Experiences of diagnosis, symptoms, and use of reliever inhalers in patients with asthma and concurrent inducible laryngeal obstruction or breathing pattern disorder: qualitative analysis of a UK asthma online community. *J Med Internet Res* 25, 1–25 (2023).

8. Castro, P. F., Larrain, G., Perez, O. & Corbalan, R. Chronic hyperventilation syndrome associated with syncope and coronary vasospasm. *Am J Med* 109, 78–80 (2000).

9. Chenivesse, C. *et al.* Severely impaired health-related quality of life in chronic hyperventilation patients: Exploratory data. *Respir Med* 108, 517–523 (2014).

10. Cherif, H. *et al.* Incidence and predictive factors of hyperventilation syndrome in patients after COVID 19 pneumonia: a prospective cohort study. *F1000 Research* 13, 1497 (2024).

11. Chevalier, B. & Schwartzstien, R. M. Hyperventilation syndrome: insights into a puzzling disorder. *The Journal of Respiratory Disease* 21, 569–574 (2000).

12. Conway, A. V., Freeman, L. J. & Nixon, P. G. Hypnotic examination of trigger factors in the hyperventilation syndrome. *Am J Clin Hypn* 30, 296–304 (1988).

13. Decuyper, M., De Bolle, M., Boone, E. & De Fruyt, F. The relevance of personality assessment in patients with hyperventilation symptoms. *Health Psychology* 31, 316–322 (2012).

14. Denton, E., Bondarenko, J. & Hew, M. Clinical characteristics of dysfunctional breathing in difficult asthma. *Respirology* 23, 174 (2018).

15. Evans, R. W. Neurologic aspects of hyperventilation syndrome. *Semin Neurol* 15, 115–125 (1995).

16. Freeman, L. F., Conway, A. & Nixon, P. G. F. Physiological responses to psychological challenge under hypnosis in patients considered to have the hyperventilation syndrome: implications for diagnosis and therapy. *J R Soc Med* 79, 76 (1986).

17. Freeman, L. J., Conway, A. V. & Nixon, P. G. F. Heart rate response, emotional disturbance and hyperventilation. *J Psychosom Res* 30, 429–436 (1986).

18. Gardner, W. N. & Bass, C. Hyperventilation in clinical practice. *Br J Hosp Med* 41, 73–81 (1989).

19. Gilbert, C. Emotional sources of dysfunctional breathing. *J Bodyw Mov Ther* 2, 224–230 (1998).

20. Goff, A. M. & Gaensler, E. A. Hyperventilation syndrome. *Respiration* 26, 359–368 (1969).

21. Greene, M. C. Functional dysphonia and the hyperventilation syndrome. *Br J Disord Commun* 19, 263–272 (1984).

22. Grossman, P. & De Swart, J. C. G. Diagnosis of hyperventilation syndrome on the basis of reported complaints. *J Psychosom Res* 28, 97–104 (1984).

23. Hagman, C., Janson, C. & Emtner, M. A comparison between patients with dysfunctional breathing and patients with asthma. *Clin Respir J* 2, 86–91 (2008).

24. Han, J. N., Stegen, K., Schepers, R., Van Den Bergh, O. & Van De Woestijne, K. P. Subjective symptoms and breathing pattern at rest following hyperventilation in anxiety and somatoform disorders. *J Psychosom Res* 45, 519–532 (1998).

25. Hegel, M. T., Abel, G. G., Etscheidt, M., Cohen-Cole, S. & Wilmer, C. L. Behavioural treatment of angina-like chest pain in patients with hyperventilation syndrome. *Journal of Behaviour Therapy and Experimental Psychiatry* 20, 31–39 (1989).

26. Hoes, M. J., Colla, P. Van, van Doorn, P. & Folgering, H. Hyperventilation and panic attacks. *J Clin Psychiatry* 48, 435–437 (1987).

27. Howell, J. B. L. Behavioural breathlessness. *Thorax* 45, 287–292 (1990).

28. Huey, S. R. & West, S. G. Hyperventilation: its relation to symptom experience and to anxiety. *J Abnorm Psychol* 92, 422–432 (1983).

29. Kang, B. S. A case of hyperventilation syndrome mimicking complex partial seizure: usefulness of EEG monitoring in emergency department. *J Epilepsy* 5, 20–22 (2015).

30. Kerr, W. J., Dalton, J. W., Gliebe, P. A. & Francisco, S. Some physical phenomena associated with the anxiety states and their relation to hyperventilation. *Ann Intern Med* 11, 961–992 (1937).

31. Kerr, W. J., Gliebe, P. A. & Dalton, J. W. Physical phenomena associated with anxiety states: the hyperventilation syndrome. *Cal West Med* 48, 12–16 (1938).

32. King, G. E. Hyperventilation syndrome simulating ischemic heart disease. *J Med Assoc Ga* 73, 177–179 (1984).

33. Koniukhovskaia, J. *et al.* The relationship between anxiety and dysfunctional breathing among the Russian population during the COVID-19 pandemic. *European Psychiatry* 65, S254–S254 (2022).

34. Lapperre, T. *et al.* Dysfunctional breathing impacts symptom burden in Chronic Obstructive Pulmonary Disease (COPD). *European Respiratory Journal* 56, 124 (2020).

35. Lazarus, H. R. & Kostan, J. J. W. Psychogenic hyperventilation and death anxiety. *Psychosomatics: Journal of Consultation and Liaison Psychiatry* 10, 14–22 (1969).

36. Loew, S. *et al.* Dysfunctional breathing after COVID-19: symptoms, functional impact and quality of life. *European Respiratory Journal* 60, 1940 (2022).

37. Magarian, G. J. Hyperventilation syndromes: infrequently recognized common expressions of anxiety and stress. *Medicine* 61, 219–236 (1982).

38. Magarian, G. J. & Olney, R. K. Absence spells: hyperventilation syndrome as a previously unrecognized cause. *Am J Med* 76, 905–909 (1984).

39. Maltinsky, W. *et al.* Illness perceptions, symptom severity and psychosocial outcomes in adults with dysfunctional breathing. *Journal of Asthma* 62, 226–235 (2025).

40. Mooney, S. & Candy, S. The real cost of effective treatment: a single case study of a patient with hyperventilation syndrome. *New Zealand Journal of Physiotherapy* 36, 88 (2008).

41. Nguyen, V. Q., Byrd, R. P. J., Fields, C. L. & Roy, T. M. DaCosta’s syndrome: chronic symptomatic hyperventilation. *J Ky Med Assoc* 90, 331–3334 (1992).

42. Noehren, T. H. Hyperventilation syndrome and its relation to pulmonary emphysema. *N Y State J Med* 66, 1076–1080 (1966).

43. Ok, J.-M., Park, Y.-B. & Id, Y.-J. P. Association of dysfunctional breathing with health-related quality of life: A cross-sectional study in a young population. *PLoS One* 13, e0205634 (2018).

44. Ostroglazov, V. G. The psychophysiological aspects of the hyperventilation syndrome. *Hum Physiol* 24, 539–546 (1998).

45. O’Sullivan, G. *et al.* Psychophysiological investigations of patients with unilateral symptoms in the hyperventilation syndrome. *British Journal of Psychiatry* 160, 664–667 (1992).

46. Peper, E., Gilbert, C. D., Harvey, R. & Lin, I.-M. Did you ask about abdominal surgery or injury? A learned disuse risk factor for breathing dysfunction. *Biofeedback* 43, 173–179 (2015).

47. Perkin, G. D. & Joseph, R. Neurological manifestations of the hyperventilation syndrome. *J R Soc Med* 79, 449–450 (1986).

48. Pincus, J. H. Disorders of conscious awareness: hyperventilation syndrome. *Br J Hosp Med* 19, 312–313 (1978).

49. Richter, J. C. Acute stress disorder in the times of COVID-19: case presentation and discussion of a young asthmatic with acute onset dysfunctional breathing syndrome. *European Journal of Allergy and Clinical Immunology* 76, 148 (2021).

50. Roberts, S. J. Hyperventilation syndrome. *J Emerg Nurs* 14, 175–179 (1988).

51. Rogers, J. Health related quality of life in patients with dysfunctional breathing referred to a cardiopulmonary exercise testing service. *European Respiratory Journal* 54, PA1116 (2019).

52. Saccomani, M. *et al.* Psychosocial morbidity but not clinical control is associated with dysfunctional breathing in moderate and severe asthmatics. *European Respiratory Journal* 44, P2194 (2014).

53. Saisch, S. G. N., Wessely, S. & Gardner, W. N. Patients with acute hyperventilation presenting to an inner-city emergency department. *Chest* 110, 952–957 (1996).

54. Sedeh, F. B. *et al.* Dysfunctional breathing in patients with severe asthma is associated with poor asthma control and low quality of life. *European Respiratory Journal* 50, (2017).

55. Sedeh, F. B. *et al.* The impact of dysfunctional breathing on the level of asthma control in difficult asthma. *Respir Med* 163, 1–7 (2020).

56. Shu, B.-C. *et al.* Parental attachment, premorbid personality, and mental health in young males with hyperventilation syndrome. *Psychiatry Res* 153, 163–170 (2007).

57. Smith, C. W. Hyperventilation syndrome: bridging the behavioural organic gap. *Postgrad Med* 78, 74–84 (1985).

58. Takeda, N. *et al.* Prevalence and characteristics of dysfunctional breathing in patients with asthma in the Japanese population. *Respir Investig* 62, 1015–1020 (2024).

59. Van Dixhoorn, J. & Duivenvoorden, H. J. Behavioural characteristics predisposing to hyperventilation complants: ‘emphasis on exhailing’ and ‘time pressure’. *Gedrag and Gezondheid: Tijdschrift voor Psychologie en Gezondheid* 14, 169–174 (1986).

60. Wheatley, C. E. Hyperventilation syndrome: a frequent cause of chest pain. *Chest* 68, 195–199 (1975).

61. Wilson, C., Harley, C. & Steels, S. How accurate is the prehospital diagnosis of hyperventilation syndrome? *Journal of Paramedic Practice* 12, 445–454 (2020).

62. Zraik, N., Boubrit, L., Randrianarivelo, O., Becquemin, M.-H. & Hatem, S. Inappropriate hyperventilation syndrome, an under recognised cause of impaired exercise capacity after cardiac surgery. *Acta Physiologica* 214, P01012 (2015).
