# Supplementary material for: Living with breathing pattern disorder: a scoping review
Source: NPJ Prim Care Respir Med. 2026 Mar 25;36:30. doi: 10.1038/s41533-026-00495-5 (PMC13181008; doi:10.1038/s41533-026-00495-5)

**Living with breathing pattern disorder: A scoping review
Catherine Moffat; Susan Walker; Jonathan Fuld; Shanlee Higgins**

**Database search strategy undertaken on the 13^th^ May 2025**

**Applied Social Sciences Index and Abstracts (ASSIA) via ProQuest database search strategy**

Limited to adults, English. No date limit.

“chronic hyperventilation” OR “hyperventilation syndrome*" OR "dysfunctional breathing" OR "breathing pattern dysfunction*" OR "disordered breathing" OR "breathing pattern disorder*" OR "idiopathic hyperventilation" – Title

OR

“chronic hyperventilation” OR “hyperventilation syndrome*" OR "dysfunctional breathing" OR "breathing pattern dysfunction*" OR "disordered breathing" OR "breathing pattern disorder*" OR "idiopathic hyperventilation" - Abstract

**
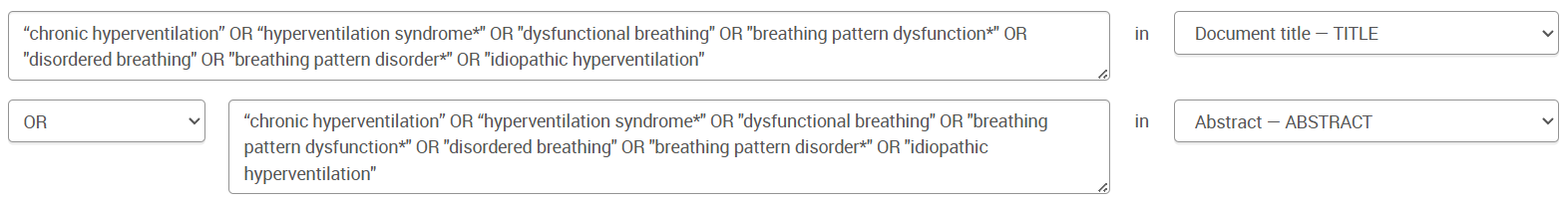
**

**Cumulative Index to Nursing and Allied Health Literature (CINAHL) via EBSCOhost database search strategy**

Limited to all adults, English. No date limit.

“chronic hyperventilation” OR “hyperventilation syndrome*" OR "dysfunctional breathing" OR "breathing pattern dysfunction*" OR "disordered breathing" OR "breathing pattern disorder*" OR "idiopathic hyperventilation" – title

OR

“chronic hyperventilation” OR “hyperventilation syndrome*" OR "dysfunctional breathing" OR "breathing pattern dysfunction*" OR "disordered breathing" OR "breathing pattern disorder*" OR "idiopathic hyperventilation" - abstract


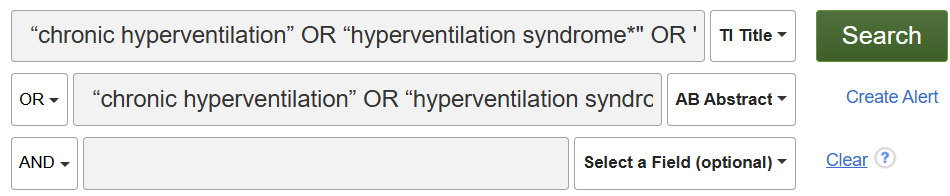


Then select tick line of results and put button search with “AND” then go to top and change to NOT put sleep.


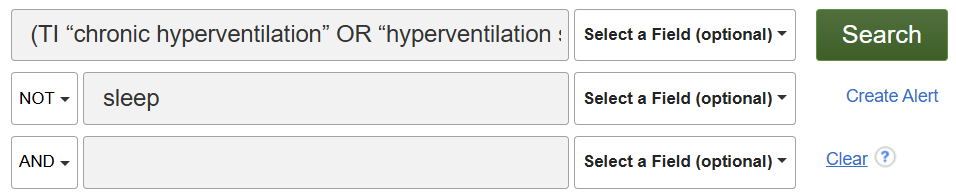


Then tick new search found and put button search with AND then go to top and change to NOT and put nocturnal.


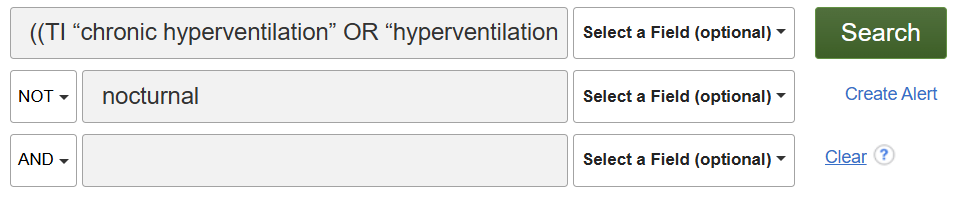


**Embase via Ovid database search strategy**

Limited to Human, English, Adults 18+ (18-64 and 65+), Medline records removed. No date limit.

(chronic ADJ hyperventilation) OR (hyperventilation ADJ syndrome$) OR (dysfunctional ADJ breathing) OR (breathing ADJ pattern ADJ dysfunction$) OR (disordered ADJ breathing) OR (breathing ADJ pattern ADJ disorder$) OR (idiopathic ADJ hyperventilation)

**
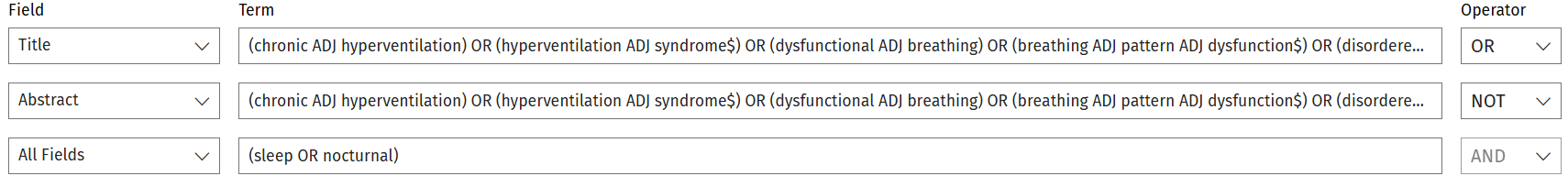
**

**Medline via EBSCOhost database search**

Limited to all adult 19+, English. No date limit.

“chronic hyperventilation” OR “hyperventilation syndrome*" OR "dysfunctional breathing" OR "breathing pattern dysfunction*" OR "disordered breathing" OR "breathing pattern disorder*" OR "idiopathic hyperventilation" – title

OR

“chronic hyperventilation” OR “hyperventilation syndrome*" OR "dysfunctional breathing" OR "breathing pattern dysfunction*" OR "disordered breathing" OR "breathing pattern disorder*" OR "idiopathic hyperventilation" – abstract


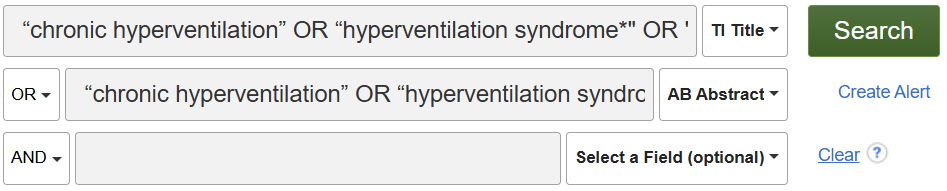


Then select tick line of results and put button search with “AND” then go to top and change to NOT put sleep.


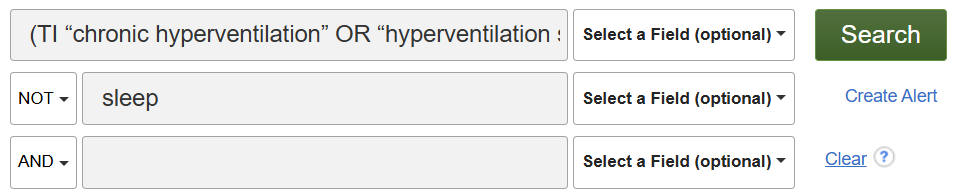


Then tick new search found and put button search with AND then go to top and change to NOT and put nocturnal.


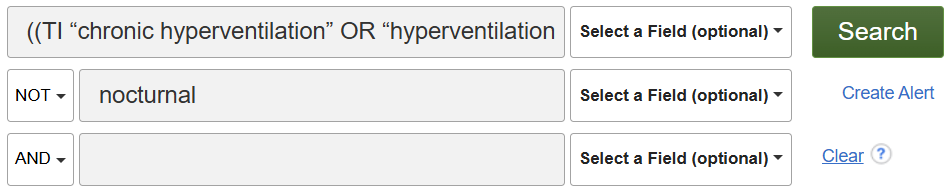


**PsycINFO via EBSCOhost search strategy**

Limited to Adults 18+, human, English. No date limit.

“chronic hyperventilation” OR “hyperventilation syndrome*" OR "dysfunctional breathing" OR "breathing pattern dysfunction*" OR "disordered breathing" OR "breathing pattern disorder*" OR "idiopathic hyperventilation" – title

OR

“chronic hyperventilation” OR “hyperventilation syndrome*" OR "dysfunctional breathing" OR "breathing pattern dysfunction*" OR "disordered breathing" OR "breathing pattern disorder*" OR "idiopathic hyperventilation" – abstract


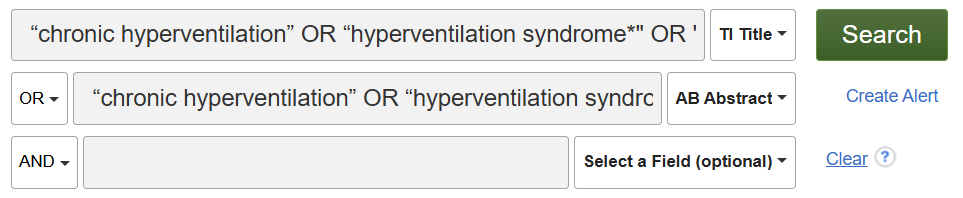


Tick results line then search with AND and change to NOT and put sleep.


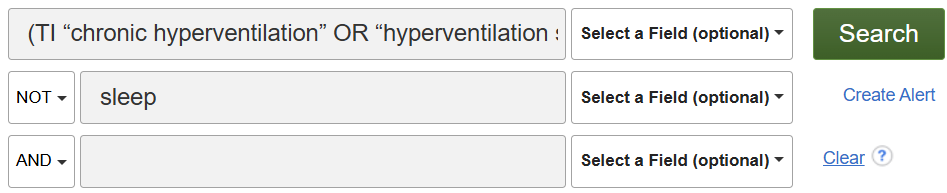


Tick these results and do above with NOT nocturnal


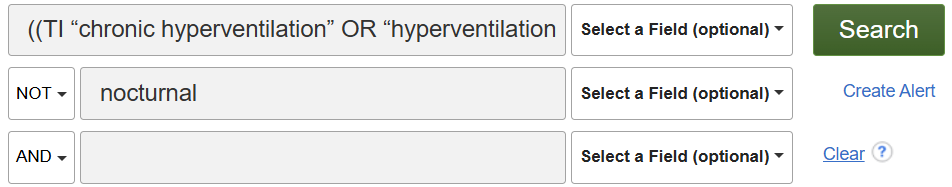

Supplement: Supplementary file 2 — Moffat BPD database search strategy v1.0 101125 [file 41533_2026_495_MOESM2_ESM.docx]
